# Quantification of hydrogen bond energy based on equations using spectroscopic, structural, QTAIM-based, and NBO-based descriptors which calibrated by the molecular tailoring approach

**DOI:** 10.1007/s00894-023-05811-1

**Published:** 2023-12-30

**Authors:** Andrei V. Afonin, Danuta Rusinska-Roszak

**Affiliations:** 1 A. E. Favorsky Irkutsk Institute of Chemistry, Siberian Division of Russian Academy of Sciences, Irkutsk, Russia; 2 Institute of Chemical Technology and Engineering, Poznan University of Technology, Poznan, Poland

**Keywords:** Intramolecular hydrogen bonds, Energy of hydrogen bonds, Hydrogen bond descriptors, QTAIM, NBO, Molecular tailoring approach

## Abstract

**Context:**

Hydrogen bonds critically influence the structure and properties of both organic molecules and biomolecules, as well as supramolecular assemblies. For this reason, the development and elaboration of methods for quantitative assessment of hydrogen bond energy is an urgent challenge. In this study, using a large series of hydroxycarbonyl aliphatic compounds with the O‒H···O = C intramolecular hydrogen bond, a bank of hydrogen bond descriptors was created, including spectroscopic, structural, QTAIM-based, and NBO-based parameters. It was shown that the O‒H vibration frequency, OH chemical shift as the spectroscopic descriptors, the O···H hydrogen bond length, O···O distance, and O‒H covalent bond length as the structural descriptors, the electron density and its Laplacian, electron potential energy density in the hydrogen bond critical point, the electron density at the ring critical point as the QTAIM-based descriptors change in a correlated manner. The same correlation is found in change of the charge transfer energy through a hydrogen bond, the occupancy of the O‒H bond antibonding orbital, the Wiberg indices of the O···H hydrogen bond, and the O‒H covalent bond, as well as the polarization of the O‒H bond, which are the NBO-based descriptors. It was also recognized that the specified descriptors from the spectroscopic, structural, QTAIM-based, and NBO-based categories are functionally related to the values of intramolecular hydrogen bond energy, quantified via the molecular tailoring approach. This allowed one to obtain a system of equations for quantitative estimation of intramolecular hydrogen bond energy based on the spectroscopic, structural, QTAIM, and NBO descriptors, which makes such quantification more dependable and reliable.

**Methods:**

To obtain the spectroscopic descriptors, the vibrational spectra and shielding constants were calculated using the GIAO method. Structural descriptors were obtained for the equilibrium geometry of molecules, calculated at the MP2(FC)/6–311 +  + (2d,2p) level using the Gaussian 09 program. The QTAIM-based descriptors were calculated using the AIMAll program within the framework of the quantum theory “Atoms in Molecules.” The NBO-based descriptors were calculated using the NBO 3.1 program implemented into Gaussian 09. To quantify the energy of intramolecular hydrogen bonds, molecular fragmentation was used within the molecular tailoring approach.

**Supplementary Information:**

The online version contains supplementary material available at 10.1007/s00894-023-05811-1.

## Introduction

Hydrogen bonding is one of the most widespread and important types of non-valence interactions. Hydrogen bonds affect the structure, physicochemical properties, spectral characteristics, and reactivity of organic molecules and biomolecules. However, the term “hydrogen bonds” describes a group of heterogeneous interactions, since the strength of hydrogen bonds varies over a wide range. It is customary to distinguish three groups of hydrogen bonds—weak (1–4 kcal/mol), moderate (4–15 kcal/mol), and strong (15–40 kcal/mol) hydrogen bonds [1]. Each category of hydrogen bonds has its own specifics, reflected in spectral and structural manifestations and different degrees of influence on the reactivity of molecules. Hence, quantification of hydrogen bond energy is an urgent challenge.

Methods for estimating the energy of intermolecular and intramolecular hydrogen bonds are fundamentally different. Assessing the *E*_HB_ energy of intermolecular hydrogen bond can be done due to Eq. (1) [2]:

1 $$E_\text{HB}=E\left(\mathrm D\mathrm o\mathrm n\;\bullet\;\mathrm A\mathrm c\mathrm c\right)-\lbrack E(\text{Don})+E(\text{Acc})\rbrack$$

where *E*(Don•Acc) is the total energy of the hydrogen-bonded complex Don•Acc, *E*(Don), and *E*(Acc) are the energy of donor molecule Don and acceptor molecule Acc, respectively.

However, Eq. (1) is not applicable to the estimation of the intramolecular hydrogen bond energy (IMHB), since the molecule cannot be divided into parts without destruction. For this reason, no clear definition of the IMHB energy is present [3, 4], but it is implied that this is an inseparable contribution to the total energy of the molecule [5]. The IMHB energy can be evaluated as the difference in the energies of two conformers, one of which is stabilized by IMHB, and in the other the IMHB is broken [6–9]. However, this method of quantification of the IMHB energy is too rough as a number of intramolecular interactions (steric, dipole–dipole, electrostatic etc.) are changed due to conversion of one conformer to another and hydrogen bonding is only one of these interactions.

A more refined method for quantitative assessment of the IMHB energy is based on establishing a functional relationship between the IMHB energy and the values of hydrogen bond descriptors in the form (2):

2 $${E}_{{\text{HB}}}=f(D)$$

where *D* is the value of hydrogen bond descriptor.

This method is named as the function-based approach (FBA) [5]. The hydrogen bond descriptors used in the FBA method are quite diverse and both the theoretical and experimental parameters can be used as hydrogen bond descriptors. Four categories of hydrogen bond descriptors can be distinguished. The experimentally measurable descriptors of the X − H⋅⋅⋅Y hydrogen bond include some spectral parameters (shift in the X − H vibration frequency in the IR spectrum [10–12] and the low-field shift of the bridging hydrogen signal in the NMR spectrum due to hydrogen bonding [13–15]) and structural parameters (the H⋅⋅⋅Y hydrogen bond length and the X − H covalent bond length determined from XRD [11, 16–18]). Theoretical descriptors are parameters calculated within the framework of the quantum theory of “Atoms in Molecules” [19] (QTAIM-based descriptors; e.g., the *ρ*_BCP_ electron density at the critical point of the hydrogen bond [20–22] and the *V*_BCP_ potential energy density at the critical point of the hydrogen bond [23–25] are widely used) and the natural bond orbitals approach [26, 27] (NBO-based descriptors; e.g., the charge transfer energies through hydrogen bond [28–30] and the occupancy of the antibonding Y − H orbital [31–33] are widely used). The QTAIM- and NBO-based descriptors should be noted to be often used together to assess the strength of non-valence interactions [34–36].

A shortcoming of the FBA method is the fact that the hydrogen bond descriptors used in this method do not have a recognized gradation and are employed in an arbitrary manner. The most commonly used descriptors are generally preferred, and the most popular is the *V*_BCP_ potential energy density at the hydrogen bond critical point due to the well-known Espinosa–Molins–Lecomte equation and its modification [37–39]. However, there is no evidence to suggest that it is a superior hydrogen bond descriptor that provides greater reliability in the IMHB energy estimation than the less popular descriptors. A comparative analysis of hydrogen bond descriptors from different categories was not yet carried out and the gradation of priority for using of descriptors to evaluate hydrogen bond energies was not established. Besides, the functional dependences of the IMHB energies on the values of the hydrogen bond descriptors within the framework of the FBA method need to be calibrated in order to obtain reasonable magnitudes of the IMHB energies [5, 11]. To calibrate the functional dependencies of the FBA method, it is necessary to have reference values of the IMHB energy obtained by another method.

An alternative method for quantifying the hydrogen bond energy with respect to the FBA is the molecular tailoring approach (MTA). The MTA method was successfully applied to quantitative estimation of the IMHB energy in medium-sized and large molecules [40–49]. The MTA method is based on the fragmentation of molecules and calculation of the hydrogen bond energy due to the energy balance in the form (3):

3 $${E}_{{\text{HB}}}=E\left({{\text{M}}}\_{{\text{AccHB}}}\right)+E\left({{\text{M}}}\_{{\text{DonHB}}}\right)-[E({\text{M}}\_{\text{IMHB}})+E({\text{M}}\_{\text{RA}})]$$

where *E*(M_AccHB) is the energy of a molecule that has an H-bond acceptor as a fragment; *E*(M_DonHB) is the energy of a molecule that has an H-bond donor as a fragment; *E*_HB_(M_IMHB) is the energy of a molecule possessing IMHB X − H⋅⋅⋅Y; *E*(M_RA) is the energy of a molecule consisting of “excess” atoms, which appear due to the imposition of molecules with an acceptor and a donor of H-bond as compared to a molecule with IMHB.

Although the MTA method provides a direct estimate of the IMHB energy, it is more complex in relation to the FBA, since fragmentation of molecules and additional calculations of the energies of molecular fragments are required within the framework of the MTA. On the other hand, as the MTA method yields a reference quantity of the hydrogen bond energy, it is suitable to calibrate the equations relating the values of hydrogen bond descriptors to the hydrogen bond energy within the framework of the FBA method.

Previously, a quantitative assessment of the O‒H···O = C IMHB energies was carried out via the MTA method in a very large series of the hydroxycarbonyl aliphatic compounds [50]. The O‒H···O = C IMHB energies in these compounds have been shown [50] to vary in the wide range from 1 to 14 kcal/mol. Therefore, this series is convenient both for the comparative analysis and ranking of various spectral, structural, QTAIM-, and NBO-based hydrogen bond descriptors and for the calibrating of the equations of the FBA method due to the availability of reference values of the IMHB energies obtained by the MTA method. Spectroscopic, structural, and partially QTAIM-based descriptors were calculated in ref. [50]. The NBO-based and additional QTAIM-based descriptors are calculated in this study.

This investigation was carried out in two stages with the goal of identifying the most reliable descriptors of the hydrogen bond and obtaining the equations that allow to quantify the IMHB energy using these descriptors. At the first stage, four categories of hydrogen bond descriptors (spectral, structural, QTAIM- and NBO-based) were formed which depend on the strength of the O‒H···O = C IMHB in the compounds under study. Then, the relationships between descriptors from different categories were established and a common bank of descriptors was created that detect the correlated changes in values with an increase or decrease in the IMHB energies. At the second stage, the system of equations was obtained that relate the values of the hydrogen bond descriptors and the values of the IMHB energies, estimated via the MTA method. In this way, the equations of the FBA method were calibrated in order to quantify the IMHB energy for other series of the hydrogen-bonded compounds. Also, a gradation was made of the preference for using the hydrogen bond descriptors to quantitative estimate the IMHB energy.

## Computational methods

**Scheme 1:**
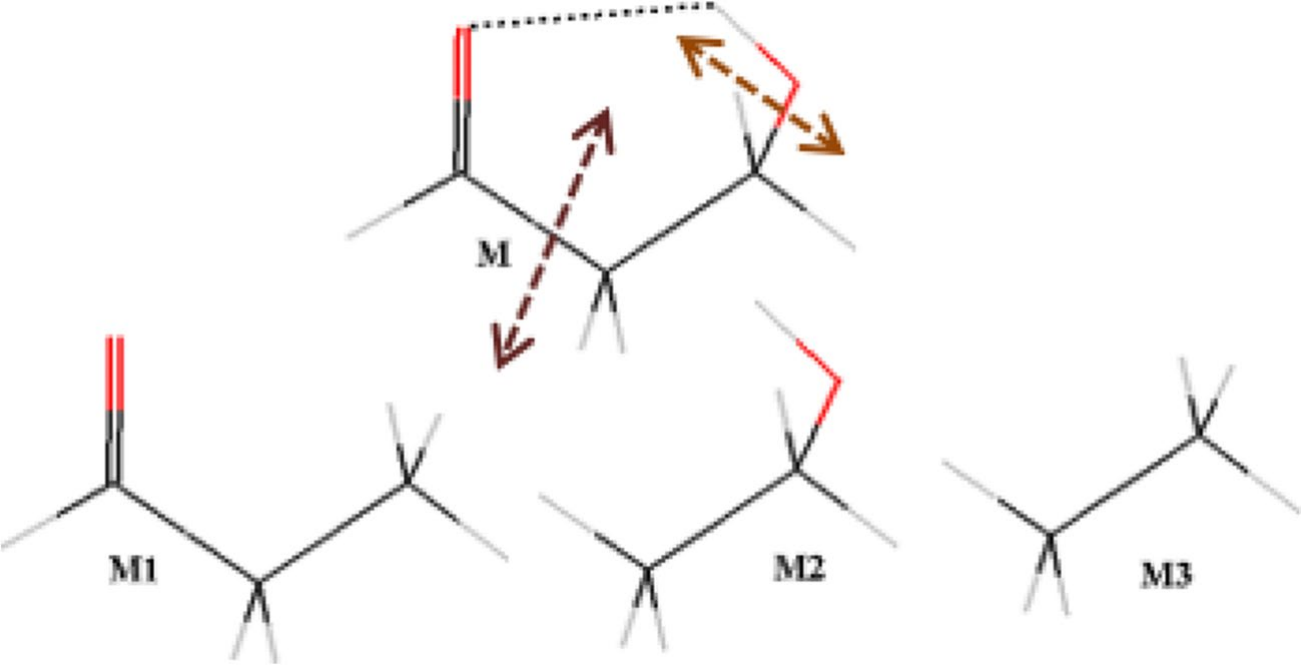
Fragmentation scheme for the *E*_HB_(MTA) value calculation on example of compound **1**

The energy of the O − H⋅⋅⋅O = C IMHB in studied compounds **1–103** was estimated [50] in accordance with the following fragmentation schemes. The entire M molecule is the main “fragment.” The M1 fragment presents the M molecule without the H-bond donor, while the M2 fragment is the M molecule without the H-bond acceptor. As the excess atoms appear when the M1 and M2 fragments are superimposed, an additional M3 fragment is introduced to compensate them (see Scheme 1). At the cutting site of the entire M molecule, the hydrogen atoms are placed at the distance of 1.1 Å from the corresponding carbon atom (see [40] and [50] for more details). The *E*_HB_(MTA) values of the IMHB energy obtained via MTA method are calculated by Eq. (4):

4 $${E}_{{\text{HB}}}\left({\text{MTA}}\right)=\left[E\left({\text{M}}\right)+E\left({\text{M}}3\right)\right]-[E({\text{M}}1)+E({\text{M}}2)]$$

where the *E*(M), *E*(M1), *E*(M2), *E*(M3) values are the energy of the entire M molecule and the M1, M2, M3 fragments, respectively.

As the IMHB make a negative contribution to the total energy of the molecule, the *E*_HB_(MTA) values are always negative. For the sake of simplifying the results discussion, we use the inverse value of the *E*_HB_(MTA) parameter from Eq. (4) throughout the article, i.e., ‒*E*_HB_(MTA) values. Thus, an increase in the ‒*E*_HB_(MTA) values corresponds to the IMHB strengthening and vice versa.

The Gaussian 09 program package [51] was used to carry out the calculations. The geometry of molecules under investigation were optimized using the MP2(FC)/6–311 +  + G(2d,2p) protocol [50]. As stated in Ref. [50], all calculated structures have no imaginary frequencies in the IR spectrum and correspond to a local energy minimum. The QTAIM-based descriptors of the studied compounds were calculated on the MP2-derived wavefunctions in Ref. [50] with the exception of the potential energy density *V*_BCP_ at the critical point of the hydrogen bond, shown for the first time in this article. The NBO-based descriptors of compounds **1–103**, calculated for the first time in this article, were obtained using the NBO 3.1 program implemented in Gaussian 09 on the MP2-derived wavefunctions.

## Results and discussion

### Presentation of studied compounds

Compounds **1–103** used for benchmark calculations of the spectroscopic, structural, QTAIM-based, and NBO-based descriptors (vide infra) are taken from Ref. 50. The full list of studied compounds **1–103** and the O‒H···O = C IMHB energy values for them quantified via MTA [‒*E*_HB_(MTA)] are presented in Tables S1 – S7 (Supplementary Information, pp. S2–S8). The values of the calculated spectroscopic, structural, QTAIM-based, and NBO-based descriptors of hydrogen bond for compounds **1–103** are given in Tables S8 and S9 (Supplementary Information, pp. S9–S16).

**Scheme 2:**
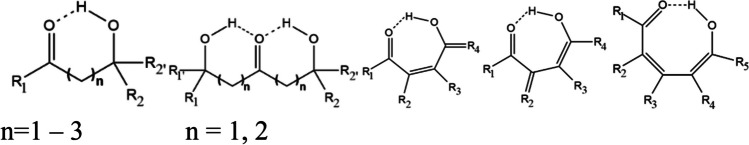
The structure of the typical studied compounds

Typical structures from the studied series of compounds are presented in Scheme 2.

Each of the studied compounds has the same O‒H···O = C IMHB. However, the structures of the studied compounds are quite diverse. In these structures, the O‒H···O = C IMHB closes a six-, seven-, and eight-membered quasi-cycle. There is a group of compounds with the O‒H···C(O)···H–O bifurcation hydrogen bond. The hydrogen bond donor and acceptor are not conjugated in the most compounds, as they are separated by an aliphatic fragment. However, there is a group of compounds in which the hydrogen bond donor and acceptor are conjugated through a system of unsaturated bonds. A variety of structures for compounds with the O‒H···O = C IMHB is necessary in order to identify general trends in the change in the NBO-based descriptors depending on the strength of the hydrogen bond, which are invariant to the specific structural features of individual molecules.

At the same time, it should be emphasized that none of the compounds studied here belongs to the class of resonance-assisted hydrogen bonds (RAHB), where the hydrogen bond donor and acceptor are separated by one double bond and an unsaturated six-membered quasi-cycle is formed. Compounds with the hydrogen bond on the aromatic platform (aromaticity-assisted hydrogen bonds, arom-AHB) are also not considered. Molecules with the RAHB and arom-AHB are a peculiar class of compounds and should be considered separately from molecules with the non-conjugated IMHB [45, 49].

### Characteristics of the O‒H···O = C hydrogen bond descriptors for the studied compounds

This article analyzes twenty descriptors that can be used to quantify the energy of the O‒H···O = C IMHB in the compounds under study. These descriptors are divided into four categories:

I. Spectroscopic descriptors
II. Structural descriptors
III. QTAIM-based descriptors
IV. NBO-based descriptors

The specification of potential hydrogen bond descriptors is given in Table 1.

**Table 1:** Specification of potential hydrogen bond descriptors of the O‒H···O = C intramolecular hydrogen bond in the studied compounds

Category of descriptor	N descriptor	Descriptor	Decryption of the descriptor
I. Spectroscopic	1	ν_O‒H_	Vibration frequency of the O‒H bond
	2	*ν*_C=O_	Vibration frequency of the C = O bond
	3	*δ*_OH_	Chemical shift of the bridging hydrogen of the O‒H group
II. Structural	4	*r*_O∙∙∙H_	Distance between the oxygen of the C = O group and the hydrogen of the O‒H group (hydrogen bond length)
	5	*r*_O∙∙∙O_	Distance between the oxygen of the C = O group and the oxygen of the O‒H group
	6	*l*_O‒H_	O‒H covalent bond length
III. QTAIM-based	7	*ρ*_BCP_	Electron density at the hydrogen bond critical point
	8	*V*_BCP_	Electron potential energy density at the hydrogen bond critical point
	9	∇^2^*ρ*	Laplacian of the electron density at the hydrogen bond critical point
	10	*ρ*_RCP_	Electron density at the ring critical point for a quasi-cycle closed by a hydrogen bond
	11	*ρ*_RCP_*	Electron density at the ring critical point for a quasi-cycle closed by a hydrogen bond, only six-membered quasi-cycle
IV. NBO-based	12	Σ(*σ* → σ*)^a^	Sum of charge transfer energies from oxygen lone pairs to the antibonding σ*(O–H) orbital through hydrogen bond
	13	*n*[σ*(O‒H)]	Occupancy of the antibonding σ*(O‒H) orbital
	14	[*n*(LP_1_) + *n*(LP_2_)]	Sum of the occupancies of the lone pair orbitals of the oxygen atom of the C = O group
	15	W(O···H)	Wiberg bond index for the O···H hydrogen bond
	16	W(O–H)	Wiberg bond index for the O‒H covalent bond
	17	W(C = O)	Wiberg bond index for the C = O hydrogen bond
	18	P(O–H)^b^	Polarization of the O–H bond
	19	*σ*-P(C = O)	Polarization of the σ-bond of the C = O group
	20	π-P(C = O)	Polarization of the π -bond of the C = O group

^a^Σ(σ → σ*) = E^(2)^[LP_1_ → σ*(O–H) + LP_2_ → σ*(O–H)] where *E*^(2)^[LP_1_ → σ*(O–H)] and E^(2)^[LP_2_ → σ*(O–H)] are the energy of charge transfer from two lone pairs of the oxygen atom of the C = O group to the antibonding σ*(O–H) orbital of the O–H bond through a hydrogen bond

^b^Polarization of the O–H bond in the framework of the NBO is defined as the square of the polarization coefficient c_O_ in expression [52]: *σ*_OH_ = c_O_h_O_ + c_H_h_H_, where *σ*_OH_ is natural hybrid orbital, h_O_ and h_H_ are bond hybrids

These four categories of hydrogen bond descriptors were chosen for the following reasons. The *ν*_O‒H_ and *δ*_OH_ spectroscopic descriptors and the *r*_O∙∙∙H_ and *r*_O∙∙∙O_ structural descriptors were used previously for a quantitative estimation of the IMHB energy [10–18, 53–55]. Therefore, they can serve as reference descriptors for comparison with descriptors from other categories. The *ρ*_BCP_ and *V*_BCP_ QTAIM-based descriptors are also well-known parameters for quantifying the IMHB energy [20–22, 37–39]. However, the suitability of a broader range of descriptors from this category for a quantitative evaluation of the IMHB strength remains uncertain. The Σ(*σ* → σ*) and *n*[σ*(O‒H)] NBO-based descriptors were used only for qualitative analysis of the IMHB strength [56–60]. The possibility of using the NBO-based descriptors for quantitative estimation of the IMHB energy was not yet recognized.

### Establishing the relationship between hydrogen bond descriptors

#### QTAIM-based descriptors versus spectroscopic and structural descriptors

To create a common bank of reliable hydrogen bond descriptors, it is necessary to establish functional relationships between descriptors from different categories. The functional interrelation was sought in two versions as the linear and polynomial ones. Initially, the relationship between the QTAIM-based and both the spectroscopic and structural descriptors was recognized. The parameters of the linear and second-order polynomial dependencies of the *ρ*_BCP_, *V*_BCP_, ∇^2^*ρ*, *ρ*_RCP_, and *ρ*_RCP_* values on the *ν*_O‒H_, *ν*_C=O_, *δ*_OH_, *r*_O∙∙∙H_, *r*_O∙∙∙O_, and *l*_O‒H_ ones are given in Tables S10 and S11, respectively (Supplementary Information, pp. S17, S18). The *r* correlation coefficients for these dependencies are collected in Table 2.

**Table 2:** The *r* correlation coefficients for the linear and second-order polynomial dependencies of the QTAIM-based descriptors of hydrogen bond on the spectroscopic and structural ones

Hydrogen bond descriptor								
QTAIM-based		Spectroscopic			Structural			Average < *r* > ^b^
		*ν*_O‒H_	*ν*_C=O_	*δ*_OH_	*r*_O∙∙∙H_	*r*_O∙∙∙O_	*l*_O‒H_	
	*N*^a^	1	2	3	4	5	6	
Linear dependencies *y* = Ax + B								
*ρ*_BCP_	1	0.958	0.493	0.900	0.951	0.943	0.955	0.941
∇^2^*ρ*	2	0.903	0.461	0.897	0.962	0.949	0.904	0.923
*V*_BCP_	3	0.961	0.512	0.891	0.926	0.936	0.959	0.935
*ρ*_RCP_	4	0.196	0.145	0.121	0.225	0.014	0.103	0.132
*ρ*_RCP_*	5	0.922	0.421	0.936	0.942	0.962	0.914	0.935
Average < *r* > ^c^		0.936	0.472	0.906	0.945	0.948	0.933	
Second-order polynomial dependencies *y* = Ax^2^ + Bx + C								
*ρ*_BCP_	1	0.971	0.567	0.922	0.993	0.976	0.959	0.964
∇^2^*ρ*	2	0.949	0.503	0.899	0.974	0.957	0.932	0.942
*V*_BCP_	3	0.968	0.585	0.924	0.985	0.981	0.960	0.964
*ρ*_RCP_	4	0.210	0.232	0.132	0.230	0.073	0.144	0.158
*ρ*_RCP_*	5	0.938	0.585	0.942	0.959	0.923	0.963	0.945
Average < *r* > ^c^		0.957	0.560	0.922	0.978	0.959	0.954	

^a^Numbering of columns and lines

^b^The average < *r* > value of the correlation coefficient for the dependencies of the QTAIM-based hydrogen bond descriptor on the spectroscopic and structural descriptors. These values are calculated from columns 1 and 3–6, as the *ν*_C=O_ descriptor is excluded from consideration (see text)

^c^The average < *r* > value of the correlation coefficient for the dependencies of the QTAIM-based hydrogen bond descriptors on the spectroscopic or structural descriptor. These values are calculated from lines 1–3 and 5, as the *ρ*_RCP_ descriptor is excluded from consideration (see text)

As can be seen from Table 2, the *r* correlation coefficient for the dependence of the *ρ*_RCP_ parameter from the QTAIM-based category on any parameter from the spectroscopic and structural categories is low in the case of both the linear and second-order polynomial dependencies (*r* ≤ 0.2, Table 2). Also, the *r* correlation coefficient is low for both the linear and second-order polynomial dependencies of any descriptor from the QTAIM-based category on the *ν*_C=O_ parameter (*r* < 0.6). This suggests that the *ρ*_RCP_ and *ν*_C=O_ parameters are inappropriate hydrogen bond descriptor and can be excluded from consideration.

Changes in other descriptors from the QTAIM-based category are, to a greater or lesser extent, cohered with changes in descriptors from the spectroscopic and structural categories. The *r* correlation coefficients vary from 0.89 and 0.90 to 0.96 and 0.99 for the linear and second-order polynomial dependencies, respectively, of the QTAIM-based descriptors on the spectral and structural ones (see Table 2). As a measure of the consistency of hydrogen bond descriptors from the QTAIM-based and the spectroscopic and structural categories, one can take the average value < *r* > of the correlation coefficient for the functional dependencies between these descriptors. The highest average < *r* > correlation coefficient of 0.941 is observed for the linear dependences of the QTAIM-based *ρ*_BCP_ parameter on the spectroscopic and structural descriptors. The < *r* > value decreases to 0.935 and 0.923 for the linear dependencies of *V*_BCP_, *ρ*_RCP_* and ∇^2^*ρ*, respectively (see Table 2). The average < *r* > correlation coefficient for the second-order polynomial dependencies reduces in the following row of the QTAIM-based descriptors: *ρ*_BCP_ = *V*_BCP_ > *ρ*_RCP_*≈∇^2^*ρ* (0.964, 0.945, 0.942, respectively, Table 2).

The highest average < *r* > correlation coefficient occurs for the linear dependencies of descriptors from the QTAIM-based category on the *r*_O∙∙∙O_ and *r*_O∙∙∙H_ structural descriptors (0.948 and 0.945, respectively). The < *r* > value decreases for dependencies on the *ν*_O‒H_, *l*_O‒H_, and *δ*_OH_ descriptors (0.936, 0.933, and 0.906, respectively, Table 2). The < *r* > value for the second-order polynomial dependences of the QTAIM-based descriptors on the spectroscopic and structural ones decreases in the following order: *r*_O∙∙∙H_ > *r*_O∙∙∙O_≈ *ν*_O‒H_≈* l*_O‒H_ > *δ*_OH_ (0.978, 0.959, 0.957, 0.954, 0.922, respectively, Table 2).

Thus, the analysis of the functional dependencies of QTAIM-based descriptors on the spectroscopic and structural ones show that the *ρ*_BCP_, *V*_BCP_, ∇^2^*ρ*, *ρ*_RCP_* parameters from the category of QTAIM-based descriptors and the *ν*_O‒H_, *δ*_OH_,* r*_O∙∙∙H_,* r*_O∙∙∙O_,* l*_O‒H_ parameters from the category of spectroscopic and structural descriptors change in a mutually consistent manner and can be combined into a common bank of descriptors for quantifying the IMHB energy within the framework of the FBA method.

#### NBO-based descriptors versus spectroscopic, structural, and QTAIM-based descriptors

At the next stage, the relationship between the NBO-based and the spectroscopic, structural, and QTAIM-based descriptors was recognized. The parameters of the linear and second-order polynomial dependencies of the Σ(*σ* → σ*), *n*[σ*(O‒H)], [*n*(LP_1_) + *n*(LP_2_)], W(O···H), W(O–H), W(C = O), P(O–H), *σ*-, and *π*-P(C = O) descriptors from the NBO-based category on the ν_O‒H_ and δ_OH_ descriptors from the spectroscopic, the *r*_O∙∙∙H_, *r*_O∙∙∙O_, and *l*_O‒H_ descriptors from the structural and the *ρ*_BCP_, *V*_BCP_, ∇^2^*ρ*, and *ρ*_RCP_* descriptors from the QTAIM-based, categories are given in Tables S12 and S13, respectively (Supplementary Information, pp. S19–S 23). The *r* correlation coefficients for these dependencies are gathered in Table 3.

**Table 3:** The* r* correlation coefficients for the linear and second-order polynomial dependencies of the NBO-based hydrogen bond descriptors on the spectroscopic, structural, and QTAIM-based ones

Hydrogen bond descriptor											
NBO-based		Spectroscopic		Structural			QTAIM-based				Average < *r* > ^b^
		*ν*_O‒H_	*δ*_OH_	*r*_O∙∙∙H_	*r*_O∙∙∙O_	*l*_O‒H_	*ρ*_BCP_	∇^2^*ρ*	*V*_BCP_	*ρ*_RCP_*	
	*N*^a^	1	2	3	4	5	6	7	8	9	
Linear dependencies *y* = Ax + B											
Σ(*σ*→σ*)	1	0.952	0.887	0.914	0.890	0.960	0.981	0.944	0.980	0.829	0.926
*n*[σ*(O‒H)]	2	0.979	0.900	0.919	0.898	0.975	0.988	0.942	0.987	0.837	0.936
[*n*(LP_1_) + *n*(LP_2_)]	3	0.693	0.810	0.752	0.665	0.715	0.732	0.727	0.703	0.623	0.713
W(O···H)	4	0.976	0.910	0.924	0.902	0.965	0.990	0.946	0.988	0.849	0.939
W(O–H)	5	0.962	0.959	0.957	0.910	0.972	0.977	0.953	0.969	0.868	0.947
W(C = O)	6	0.729	0.734	0.664	0.650	0.757	0.720	0.710	0.708	0.675	0.705
P(O–H)	7	0.946	0.965	0.953	0.907	0.965	0.964	0.944	0.950	0.917	0.946
σ-P(C = O)	8	0.267	0.295	0.246	0.287	0.305	0.291	0.285	0.297	0.036	0.257
π-P(C = O)	9	0.620	0.661	0.629	0.567	0.639	0.635	0.638	0.609	0.646	0.627
Average < *r* > ^c^		0.963	0.924	0.933	0.901	0.967	0.980	0.946	0.975	0.860	
Second-order polynomial dependencies *y* = Ax^2^ + Bx + C											
Σ(*σ*→σ*)	1	0.958	0.947	0.987	0.955	0.960	0.987	0.975	0.982	0.973	0.969
*n*[σ*(O‒H)]	2	0.983	0.952	0.992	0.960	0.975	0.993	0.973	0.988	0.971	0.976
[*n*(LP_1_) + *n*(LP_2_)]	3	0.768	0.810	0.756	0.677	0.762	0.749	0.727	0.720	0.650	0.735
W(O···H)	4	0.983	0.957	0.993	0.960	0.977	0.993	0.975	0.988	0.967	0.977
W(O–H)	5	0.993	0.969	0.985	0.944	0.987	0.979	0.960	0.975	0.947	0.971
W(C = O)	6	0.747	0.750	0.710	0.695	0.759	0.722	0.726	0.709	0.687	0.723
P(O–H)	7	0.991	0.968	0.969	0.925	0.987	0.971	0.946	0.961	0.937	0.962
σ-P(C = O)	8	0.309	0.301	0.301	0.289	0.310	0.296	0.316	0.297	0.036	0.273
π-P(C = O)	9	0.665	0.665	0.637	0.587	0.662	0.638	0.638	0.612	0.657	0.640
Average < *r* > ^c^		0.982	0.959	0.985	0.949	0.977	0.985	0.966	0.979	0.959	

^a^Numbering of columns and lines

^b^The average < *r* > value of the correlation coefficient for the dependencies of the NBO-based hydrogen bond descriptor on the spectroscopic, structural and QTAIM-based descriptors. These values are calculated from columns 1–9

^c^The average < *r* > value of the correlation coefficient for the dependencies of the NBO-based hydrogen bond descriptors on the spectroscopic or structural or QTAIM-based-descriptor. These values are calculated from lines 1, 2, 4, 5, and 7, as the [*n*(LP_1_) + *n*(LP_2_)], W(C = O), *σ*-, and* π*-P(C = O) descriptors are excluded from consideration (see text)

As follows from Table 3, the *r* correlation coefficient for the dependencies of the [*n*(LP_1_) + *n*(LP_2_)], W(C = O), *σ*-, and *π*-P(C = O) parameters from the NBO-based category on any parameter from the QTAIM-based, spectroscopic, and structural categories is quite low in the case of both the linear and second-order polynomial dependencies (*r* ≤ 0.7, Table 3). This implies that the indicated parameters from the NBO-based category are poor hydrogen bond descriptors and can be excluded from consideration.

Most of the linear dependences of the Σ(*σ* → σ*), *n*[σ*(O‒H)], W(O···H), W(O–H), and P(O–H) parameters from the category of the NBO-based descriptors on the QTAIM-based, spectroscopic, and structural ones have a rather high *r* correlation coefficient above 0.94 (Table 3). However, there are a few exceptions that are worth noting. The *r* correlation coefficient is relatively poor for the linear dependencies of the Σ(*σ* → σ*), *n*[σ*(O‒H)], and W(O–H) NBO-based descriptors on the *ρ*_RCP_*, *δ*_OH_, and *r*_O∙∙∙O_ (0.829, 0.887, and 0,890; 0.837, 0.900, and 0.898; 0.849, 0.910, and 0.902), dependencies of the W(O···H) on the *ρ*_RCP_* and *r*_O∙∙∙O_ (0.868 and 0.902), dependency of the P(O–H) on the *r*_O∙∙∙O_ (0.907, Table 3). However, the *r* correlation coefficient increases significantly on going to the second-order polynomial dependencies between the noted descriptors. The *r* correlation coefficients for the second-order polynomial dependencies of the Σ(*σ* → σ*), *n*[σ*(O‒H)], W(O···H), W(O–H), and P(O–H) parameters from the category of NBO-based descriptors on the QTAIM-based, spectroscopic, and structural ones lie in the range of 0.947–0.987, 0.952–0.993, 0.957–0.993, 0.944–0.993, and 0.925–0.991, respectively (Table 3). The average < *r* > correlation coefficients of the dependencies above are quite high (0.962–0.977), and they slightly decrease in the row of descriptors W(O···H) ≈ *n*[σ*(O‒H)] > W(O–H) ≈ Σ(*σ* → σ*) > P(O–H) (0.977, 0.976, 0.971, 0.969, 0.962, respectively, Table 3).

The most reliable are the polynomial dependencies of the NBO-based descriptors on the *ρ*_BCP_ and *V*_BCP_ QTAIM-based, the *ν*_OH_ spectroscopic and the *r*_O∙∙∙H_, *l*_O‒H_ structural descriptors (< *r* >  = 0.985, 0.979, 0.982, 0.985, 0.977, Table 3). The average < *r* > correlation coefficient lowers slightly for the dependences of the NBO-based descriptors on the ∇^2^*ρ*, *ρ*_RCP_*, *δ*_OH_, and *r*_O∙∙∙O_ (0.966, 0.959, 0.959, and 0.949, Table 3).

In summary, the analysis of the functional dependencies of descriptors from the spectroscopic, structural, QTAIM-, and NBO-based categories allows one to identify those from them that are interrelated and correlated. A total of 14 such descriptors were identified—two from the spectroscopic (*ν*_O‒H_ and *δ*_OH_), three from the structural (*r*_O∙∙∙H_, *r*_O∙∙∙O_, and *l*_O‒H_), four from the QTAIM-based (*ρ*_BCP_, *V*_BCP_, ∇^2^*ρ*, and *ρ*_RCP_*), and five from the NBO-based [Σ(*σ* → σ*), *n*[σ*(O‒H)], W(O···H), W(O–H), and P(O–H)] categories. These parameters form the hydrogen bond descriptor bank summarized in Table 4. Each of these descriptors, depending on the situation, can be used to quantify the IMHB energy within the framework of the FBA method, if the equations connecting the values of the descriptors with the values of the IMHB energies are obtained.

**Table 4:** The set of the hydrogen bond descriptors from the spectroscopic, structural, QTAIM-based and NBO-based categories

Spectroscopic			Structural				QTAIM-based					
*ν*_O‒H_	*δ*_OH_		*r*_O∙∙∙H_	*r*_O∙∙∙O_		*l*_O‒H_	*ρ*_BCP_		∇^2^*ρ*	*V*_BCP_		*ρ*_RCP_*
NBO-based												
Σ(*σ* → σ*)		*n*[σ*(O‒H)]			W(O···H)			W(O–H)			P(O–H)	

#### Establishing the relationship between hydrogen bond descriptors and energy of the O‒H···O = C intramolecular hydrogen bond quantified via molecular tailoring approach

The MTA method yields a direct estimation of the IHMB energy due to Eq. (4) as the ‒*E*_HB_(MTA) parameter. To recognize which of the hydrogen bond descriptors are better cohered with the ‒*E*_HB_(MTA) parameter, the linear and second-order polynomial dependences of the descriptors from the spectroscopic, structural, QTAIM-based, and NBO-based categories on the ‒*E*_HB_(MTA) energy values were obtained. The parameters of these dependencies are given in Table S14 (Supplementary Information, p. S23), while the values of the r correlation coefficients for these dependencies are presented in Table 5.

**Table 5:** The *r* correlation coefficients for the linear and second-order polynomial dependencies of the spectroscopic, structural, QTAIM-based, and NBO-based hydrogen bond descriptors on the ‒*E*_HB_(MTA) IMHB energy

Type of descriptor	Descriptor	Correlation coefficient *r*		Δ*r*
		Linear	Second-order polynomial	
Spectroscopic	*ν*_O‒H_	0.960	0.962	0.002
	*ν*_C=O_	0.587	0.644	0.057
	*δ*_OH_	0.892	0.926	0.034
Structural	*r*_O∙∙∙H_	0.854	0.888	0.034
	*r*_O∙∙∙O_	0.819	0.821	0.002
	*l*_O‒H_	0.964	0.964	0
QTAIM-based	*ρ*_BCP_	0.916	0.917	0.001
	∇^2^*ρ*	0.869	0.883	0.014
	*V*_BCP_	0.917	0.917	0
	*ρ*_RCP_	0.157	0.230	0.073
	*ρ*_RCP_*	0.864	0.873	0.009
NBO-based	Σ(*σ* → σ*)	0.917	0.917	0
	*n*[σ*(O‒H)]	0.939	0.940	0.001
	[*n*(LP_1_) + *n*(LP_2_)]	0.652	0.714	0.062
	W(O···H)	0.938	0.938	0
	W(O–H)	0.938	0.948	0.01
	W(C = O)	0.757	0.765	0.008
	P(O–H)	0.928	0.944	0.016
	*σ*-P(C = O)	0.328	0.350	0.022
	*π*-P(C = O)	0.622	0.648	0.026

As can be seen from Table 5, the *r* correlation coefficients for both the linear and second-order polynomial dependences of the *ν*_C=O_, *ρ*_RCP_, [*n*(LP_1_) + *n*(LP_2_)], W(C = O), *σ*-, and *π*-P(C = O) descriptors on the ‒*E*_HB_(MTA) are poor (*r* < 0.8). These descriptors should be recognized as unsuitable for quantifying the IMHB energy and excluded from consideration. As for other descriptors, it is necessary to determine whether it is enough to use the linear dependencies on the ‒*E*_HB_(MTA), or whether it is necessary to pass to the second-order polynomial ones.

The *r* correlation coefficient does not change or changes negligibly on going from the linear dependencies of the *ν*_O‒H_, *l*_O‒H_, *r*_O∙∙∙O_, *ρ*_BCP_, *V*_BCP_, Σ(*σ* → σ*), *n*[σ*(O‒H)], and W(O···H) parameters on the ‒*E*_HB_(MTA) to the second-order polynomial ones (Δ*r* ≤ 0.002, Table 5). Therefore, the relationship between descriptors above and the IMHB energy can be described by a linear function. However, one can observe a noticeable increase in the *r* correlation coefficient when passing from the linear dependencies of the *δ*_OH_, *r*_O∙∙∙H_, ∇^2^*ρ*, *ρ*_RCP_*, W(O–H), and P(O–H) parameters on the ‒*E*_HB_(MTA) to the second-order polynomial ones (Δ*r* = 0.009–0.034, Table 5), i.e., the relationship between these descriptors and the IMHB energy is non-linear.

Both the linear and second-order polynomial dependencies of the *r*_O∙∙∙H_, *r*_O∙∙∙O_, ∇^2^*ρ*, and *ρ*_RCP_* parameters on the—*E*_HB_(MTA) have a rather low *r* correlation coefficient (0.8 < *r* < 0.9, Table 5). This means that the use of these descriptors to quantify the IMHB energy may be associated with significant error. The best descriptors of hydrogen bond are the *ν*_O‒H_, *δ*_OH_, *l*_O‒H_, *ρ*_BCP_, *V*_BCP_, Σ(*σ* → σ*), *n*[σ*(O‒H)], W(O···H), W(O–H), and P(O–H) parameters, since the *r* correlation coefficient for the linear or second-order polynomial dependencies of the listed descriptors on the ‒*E*_HB_(MTA) is higher than 0.9 (see Table 5). The reliability of the discussed descriptors for quantifying the IMHB energy decreases in the order: *l*_O‒H_≈ *ν*_O‒H_ > W(O–H) > P(O–H) > *n*[σ*(O‒H)] ≈ W(O···H) > *δ*_OH_ > *ρ*_BCP_ = *V*_BCP_ = Σ(*σ* → σ*) > *r*_O∙∙∙H_ > ∇^2^*ρ* > *ρ*_RCP_* > *r*_O∙∙∙O_ (maximum *r* correlation coefficient for dependencies on the ‒*E*_HB_(MTA) are 0.964, 0.962, 0.948, 0.944, 0.940, 0.938, 0.926, 0.917, 0.917, 0.917, 0.888, 0.873, 0.821, relatively, Table 5).

#### Deriving equations to quantify intramolecular hydrogen bond energy using different categories of hydrogen bond descriptors

In the previous section, the 14 hydrogen bond descriptors from the spectroscopic, structural, QTAIM-based, and NBO-based categories were identified, which somehow exhibit correlated changes depending on the IMHB strength. Bearing this in the mind, it is possible to obtain a system of equations which allows one to quantitative estimate the IMHB energy using these descriptors within the framework of the FBA method (vide supra).

Linear Eqs. (5)–(18) relating the values of the spectroscopic, structural, QTAIM-, and NBO-based hydrogen bond descriptors with the ‒*E*_HB_(MTA) O‒H···O = C IMHB energy in the studied compounds are collected in Table 6. Second-order polynomial dependences (19)–(25) of some from these descriptors on the ‒*E*_HB_(MTA) IMHB energy are gathered in Table 7. Simpler linear dependences (5), (9), (10), (12), (14)–(16) of the *ν*_O‒H_, *l*_O‒H_, *ρ*_BCP_, *V*_BCP_, Σ(*σ* → σ*), *n*[σ*(O‒H)], and W(O···H) descriptors on the ‒*E*_HB_(MTA) are more preferable than the second-order polynomial ones, as the *r* correlation coefficients are almost the same for both type of dependencies (vide supra). However, second-order polynomial dependencies (19)–(25) better describe the relationship between the *δ*_OH_, *r*_O∙∙∙H_, *r*_O∙∙∙O_, ∇^2^*ρ*, *ρ*_RCP_*, W(O–H), and P(O–H) descriptors and the ‒*E*_HB_(MTA) IHMB energy compared to linear dependencies (6)–(8), (11), (13), (17), (18) as the *r* correlation coefficients of the latter dependencies are noticeably higher than those of the former ones.

**Table 6:** Linear Eqs. (5)–(18) relating the values of the spectroscopic, structural, QTAIM-based, and NBO-based hydrogen bond descriptors with the ‒*E*_HB_(MTA) IMHB O‒H···O = C energy

Type of descriptor	Descriptor	*N* equation	Equation
Spectroscopic	*ν*_O‒H_	5	‒*E*_HB_(MTA) = − 0.010 × *ν*_O‒H_ + 41.28; *r* = 0.960
	*δ*_OH_	6	‒*E*_HB_(MTA) = 0.58 × *δ*_OH_ + 0.86; *r* = 0.892
Structural	*r*_O∙∙∙H_	7	‒*E*_HB_(MTA) = − 11.73 × *r*_O∙∙∙H_ + 27.51; *r* = 0.854
	*r*_O∙∙∙O_	8	‒*E*_HB_(MTA) = − 19.7 × *r*_O∙∙∙O_ + 59.45; *r* = 0.819
	*l*_O‒H_	9	‒*E*_HB_(MTA) = 253.37 × *l*_O‒H_ ‒ 241.51; *r* = 0.964
QTAIM-based	*ρ*_BCP_	10	‒*E*_HB_(MTA) = 192.0 × *ρ*_BCP_ ‒ 0.70; *r* = 0.916
	∇^2^*ρ*	11	‒*E*_HB_(MTA) = 70.5 × ∇^2^*ρ* ‒ 2.38; *r* = 0.869
	*V*_BCP_	12	‒*E*_HB_(MTA) = − 172.5 × *V*_BCP_ + 0.33; *r* = 0.919
	*ρ*_RCP_*	13	‒*E*_HB_(MTA) = 953.9 × *ρ*_RCP_* ‒ 10.46;* r* = 0.858
NBO-based	Σ(*σ* → σ*)	14	‒*E*_HB_(MTA) = 0.19 × Σ(*σ* → σ*) + 2.42; *r* = 0.917
	*n*[σ*(O‒H)]	15	‒*E*_HB_(MTA) = 148.4 × *n*[σ*(O‒H)] + 1.39; *r* = 0.939
	W(O···H)	16	‒*E*_HB_(MTA) = 96.8 × W(O···H) + 2.07; *r* = 0.938
	W(O–H)	17	‒*E*_HB_(MTA) = -57.3 × W(O–H) + 45.1; *r* = 0.938
	P(O–H)	18	‒*E*_HB_(MTA) = 1.65 × P(O–H) ‒ 121.2; *r* = 0.928

**Table 7:** Second-order polynomial Eqs. (19)–(25) relating the values of spectroscopic, structural, QTAIM-based, and NBO-based hydrogen bond descriptors with the ‒*E*_HB_(MTA) IMHB O‒H···O = C energy

Type of descriptor	Descriptor	N equation	Equation
Spectroscopic	*δ*_OH_	19	‒*E*_HB_(MTA) = 0.057 × *δ*_OH_^2^ ‒ 0.30 × *δ*_OH_ + 3.19; *r* = 0.936
Structural	*r*_O∙∙∙H_	20	‒*E*_HB_(MTA) = 22.50 × *r*_O∙∙∙H_^2^ ‒ 97.54 × *r*_O∙∙∙H_ + 108.29; *r* = 0.918
	*r*_O∙∙∙O_	21	‒*E*_HB_(MTA) = 51.73 × *r*_O∙∙∙O_^2^ ‒ 304.77 × *r*_O∙∙∙O_ + 450.02; *r* = 0.895
QTAIM-based	∇^2^*ρ*	22	‒*E*_HB_(MTA) = 573.7 × ∇^2^*ρ*^2^ ‒ 59.9 × ∇^2^*ρ* + 4.24; *r* = 0.900
	*ρ*_RCP_*	23	‒*E*_HB_(MTA) = 119,927 × *ρ*_RCP_*^2^ ‒ 2979.3 × *ρ*_RCP_* + 20.98; *r* = 0.900
NBO-based	W(O–H)	24	‒*E*_HB_(MTA) = 224.1 × W(O–H)^2^‒363.3 × W(O–H) + 148.92;* r* = 0.952
	P(O–H)	25	‒*E*_HB_(MTA) = 0.24 × P(O–H)^2^ ‒ 34.8 × P(O–H) + 1277.5; *r* = 0.950

**Fig. 1:**
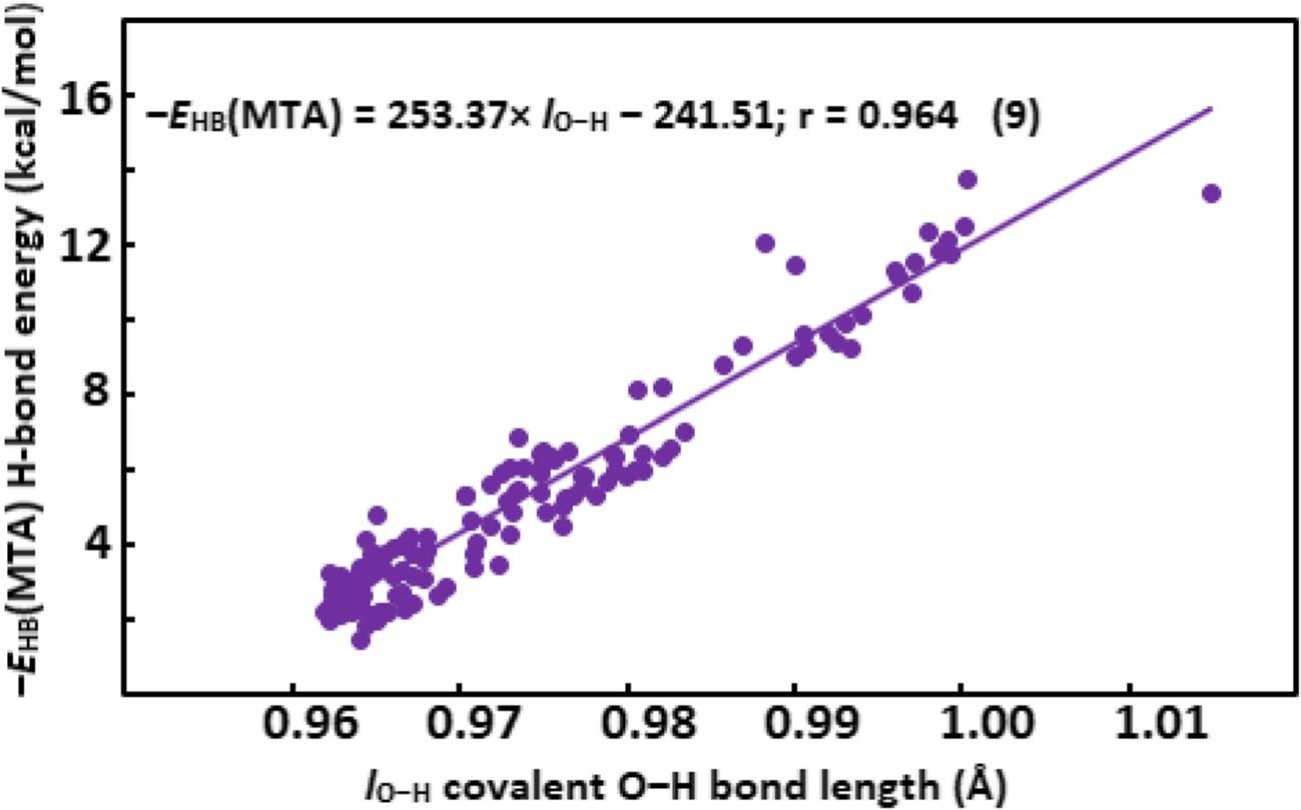
Dependence of the ‒*E*_HB_(MTA) hydrogen bond energy on the *l*_O‒H_ covalent O‒H bond length

Some of the spectroscopic, structural, and QTAIM-based descriptors were previously used to quantify the IMHB energy. Hence, a comparison of the present data with those obtained earlier should be done. Similar linear equations relating the values of the *ν*_O‒H_ and *δ*_OH_ spectral descriptors, as well as the *ρ*_BCP_ and *V*_BCP_ QTAIM-based descriptors with the IMHB energy were obtained in the ref. 11, 13, and 39, respectively. At the same time, the relationship between the *r*_O∙∙∙H_ and *r*_O∙∙∙O_ structural descriptors and the IMHB energy was described by a more complex exponential function [18, 53–55]. The *l*_O‒H_ structural descriptor, the ∇^2^*ρ* and *ρ*_RCP_* QTAIM-based descriptors were not previously used for a quantitative estimation of the IMHB energy. The best of them is the *l*_O‒H_ descriptor, since it is related to the values of the IMHB energies by a simple linear relationship (9) with a high *r* correlation coefficient (see Fig. 1).

**Fig. 2:**
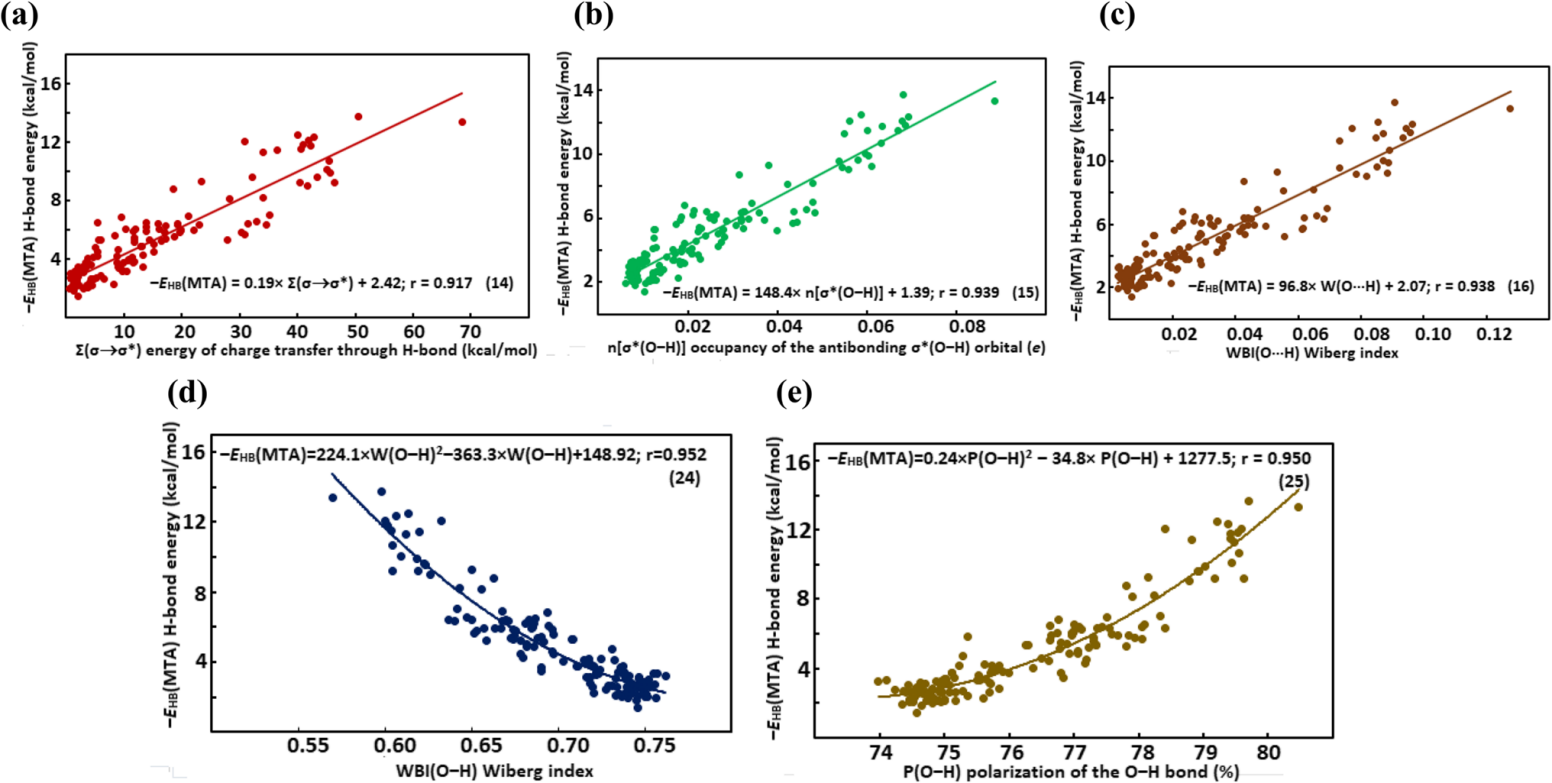
Dependence of the ‒*E*_HB_(MTA) hydrogen bond energy on the E.^(2)^[Σ(*σ* → σ*)] energy of charge transfer through hydrogen bond (**a**); the *n*[σ*(O‒H)] occupancy of the antibonding σ*(O‒H) orbital (*e*) (**b**); the WBI(O···H) Wiberg indices (**c**); the WBI(O‒H) Wiberg indices (**d**); and the P(O‒H) polarization of the O‒H bond (**e**)

Particularly noteworthy is the category of the NBO-based descriptors. When quantifying the IMHB energy, clear preference is given to the QTAIM-based parameters [61], since the NBO-based descriptors were only used to qualitatively assess the IMHB strength trends. For instance, an increase in the Σ(*σ* → σ*) and *n*[σ*(O‒H)] NBO parameters was considered as evidence of hydrogen bond strengthening [56–60]. However, the NBO-based descriptors can be used to quantitative estimate the energies of IMHB and other non-valency interactions. The firstly recognized quantitative dependences of the IMHB energies on the NBO-based parameters are shown in Fig. 2a, b, c, d, e. The inclusion of NBO-based parameters in the bank of hydrogen bond descriptors significantly increase the ability of the FBA method to quantify the IMHB energies due to additional calculations of the NBO-based descriptors within the framework of the NBO method. As can be seen from Figs. 1 and 2, using a single equation and descriptor can result in noticeable error in the quantitative estimation of hydrogen bond energy. However, the use of a system of equations with multiple descriptors allows one to minimize the error by averaging the energy values and obtain a more reliable quantitative estimate [5, 22, 39].

## Conclusions

Using a large series of compounds with the O‒H···O = C intramolecular hydrogen bond, a bank of hydrogen bond descriptors was formed, which includes descriptors from the spectroscopic, structural, QTAIM-based, and NBO-based categories. The bank includes the ν_O‒H_ vibrational frequency of the O‒H bond and the *δ*_OH_ chemical shift of the O‒H group hydrogen as the spectroscopic descriptors, the *r*_O∙∙∙H_ hydrogen bond length, the *r*_O∙∙∙O_ distance between oxygen atoms and the *l*_O‒H_ length of the O‒H covalent bond as structural descriptors. The QTAIM-based descriptors are the *ρ*_BCP_ electron density at the hydrogen bond critical point, the ∇^2^*ρ* the Laplacian of the electron density at this point, the *V*_BCP_ electron potential energy density at the hydrogen bond critical point, and the *ρ*_RCP_* electron density at the ring critical point for cycles of the same size. The NBO-based category consists of the Σ(*σ* → σ*) energy of charge transfer through a hydrogen bond, the *n*[σ*(O‒H)] occupancy of the antibonding orbital of the O‒H bond, the W(O···H) and W(O–H) Wiberg indices for the O···H hydrogen bond and O‒H covalent bond, respectively, and the P(O–H) polarization of the O–H bond. The indicated descriptors exhibit correlated changes as the O‒H···O = C intramolecular hydrogen bonding strengthens or weakens.

The descriptors above show a correlation with the O‒H···O = C intramolecular hydrogen bond energy values quantified via the molecular tailoring approach. This allows one to obtain a system of equations relating the energy of intramolecular hydrogen bonds with the values of descriptors from spectroscopic, structural, QTAIM-based and NBO-based categories. The dependencies of the intramolecular hydrogen bond energy on the ν_O‒H_ spectral, the *l*_O‒H_ structural, the *ρ*_BCP_ and *V*_BCP_ QTAIM-based and the Σ(*σ* → σ*), *n*[σ*(O‒H)], and W(O···H) NBO-based descriptors are linear. The dependencies of the intramolecular hydrogen bond energy on the δ_OH_ spectral, the *r*_O∙∙∙H_ and *r*_O∙∙∙O_ structural, the ∇^2^*ρ*, and *ρ*_RCP_* QTAIM-based, the W(O–H) and P(O–H) NBO-based descriptors are obtained in the form of a second-order polynomial.

Particular attention should be paid to the Σ(*σ* → σ*), *n*[σ*(O‒H)], W(O···H), W(O–H), and P(O–H) NBO-based descriptors. Descriptors from this category were previously used only to recognize qualitative trends in changes in the intramolecular hydrogen bonds strength. However, the data from the present study suggest that the NBO-based descriptors can be successfully used to quantify the energy of intramolecular hydrogen bonds.

Creating a bank of hydrogen bond descriptors from the four designated categories and obtaining the functional dependences of intramolecular hydrogen bond energy on the values of these descriptors significantly increases the capabilities of the functional-based approach for quantitative estimation of intramolecular hydrogen bond energy. A system of equations with multiple descriptors for quantifying the hydrogen bond energy provides a more reliable quantitative estimation and minimizes error compared to a single equation and descriptor.

### Supplementary Information

Below is the link to the electronic supplementary material.

Supplementary file1 (DOCX 580 KB)

## Data Availability

All data discussed and analyzed data contained in this published article and additional information file.
