# Supplementary material for: Quantification of hydrogen bond energy based on equations using spectroscopic, structural, QTAIM-based, and NBO-based descriptors which calibrated by the molecular tailoring approach
Source: J Mol Model. 2023 Dec 30;30(1):18. doi: 10.1007/s00894-023-05811-1 (PMC10757697; doi:10.1007/s00894-023-05811-1)
Supplement: Supplementary file 1 — Supplementary file1 (DOCX 580 KB) [file 894_2023_5811_MOESM1_ESM.docx]

**Supplementary Information:**

**Quantification of hydrogen bond energy based on equations using spectroscopic, structural, QTAIM-based and NBO-based descriptors which calibrated by the molecular tailoring approach**

**Andrei V. Afonin,^1^** **Danuta Rusinska-Roszak^2^**

^1^A. E. Favorsky Irkutsk Institute of Chemistry, Siberian Division of Russian Academy of Sciences, Irkutsk, Russia. E-mail: [andvalaf@irioch.irk.ru](https://e.mail.ru/compose?To=andvalaf@irioch.irk.ru)

^2^Institute of Chemical Technology and Engineering, Poznan University of Technology, Poznan, Poland. E-mail: [danuta.rusinska-roszak@put.poznan.pl](mailto:danuta.rusinska-roszak@put.poznan.pl)

| **Contents** | **Page** |
| --- | --- |
| **Tables S1 – S7.** Structures of studied compounds **1 – 103** and the values of ‒*E*_HB_ MTA energy of the O‒H∙∙∙O=C intramolecular hydrogen bond. | S2 – S8 |
| **Table S8.** The values of the spectroscopic, structural and QTAIM-based descriptors of hydrogen bond for compounds **1** – **103**. | S9 – S11 |
| **Table S9.** The values of the NBO-based descriptors for compounds **1**–**103**. | S12 – S16 |
| **Table S10.** The parameters of the linear dependencies of the QTAIM-based descriptors on the spectroscopic and structural descriptors. | S17 |
| **Table S11.** The parameters of the second order polynomial dependencies of the QTAIM-based descriptors on the spectroscopic and structural descriptors. | S18 |
| **Table S12.** The parameters of the linear dependencies of the NBO-based descriptors on the spectroscopic, structural and QTAIM-based descriptors. | S19 – S21 |
| **Table S13.** The parameters of the second order polynomial dependencies of the NBO-based descriptors on the spectroscopic, structural and QTAIM-based descriptors. | S21 – S23 |
| **Table S14.** The parameters of the linear dependencies and the second order polynomial dependencies of the spectroscopic, structural, QTAIM-based and NBO-based descriptors on the hydrogen bond energy estimated via molecular tailoring approach. | S23 |

**Table S1**. Structure of **1-non-RAHB – 43-non-RAHB** compounds. Taken from Ref.50.


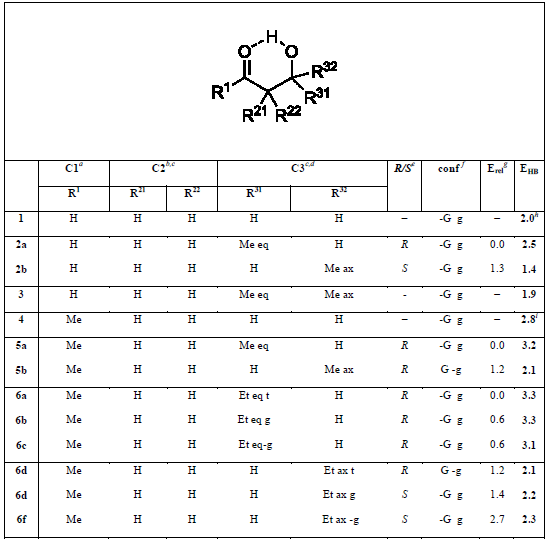

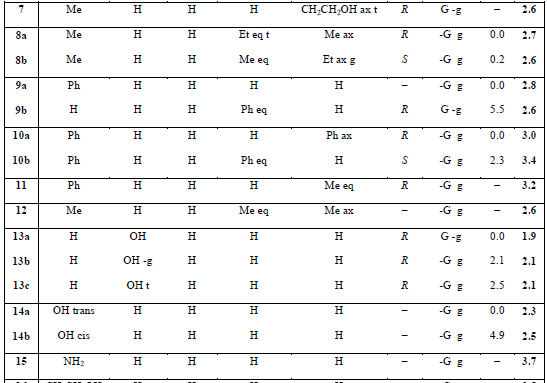


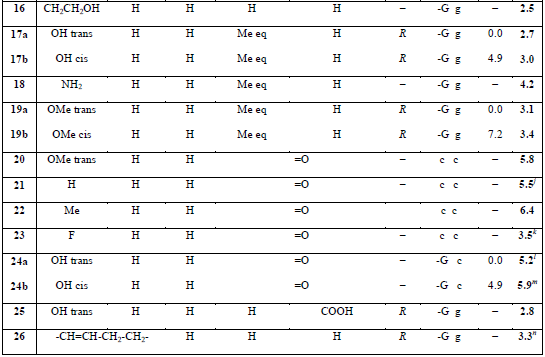

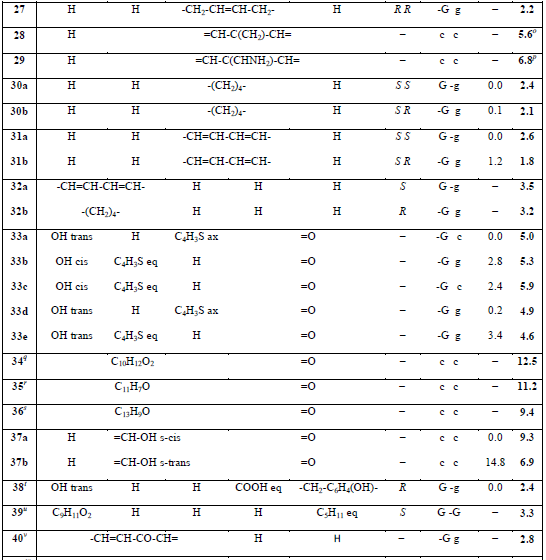


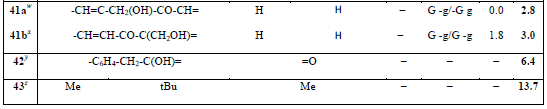


**Table S2**. Structure of **44-non-RAHB – 59-non-RAHB** compounds. Taken from Ref.50.


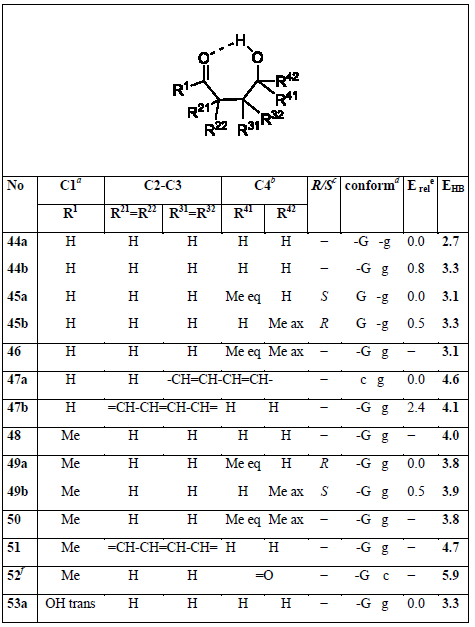

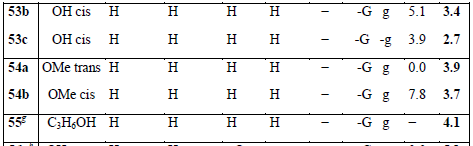


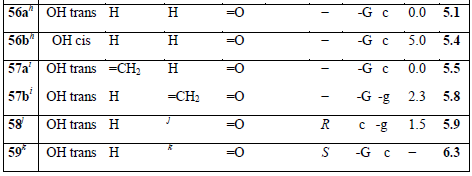


**Table S3**. Structure of **60-non-RAHB – 69-non-RAHB** compounds. Taken from Ref.50.


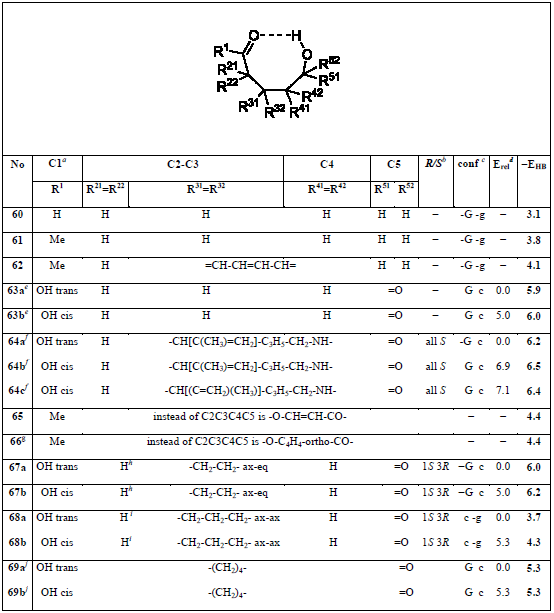


**Table S4**. Structure of **70-non-RAHB – 78-non-RAHB** compounds. Taken from Ref.50.


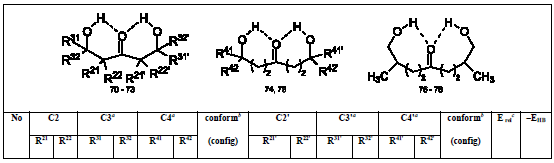

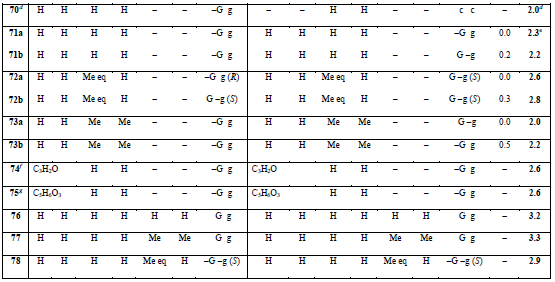


**Table S5**. Structure of **79-non-RAHB – 88-non-RAHB** compounds. Taken from Ref.50.


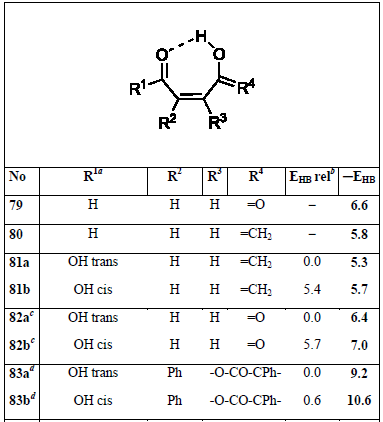


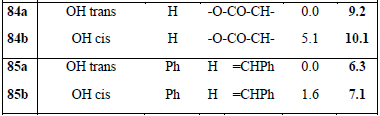

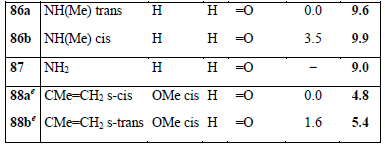


**Table S6**. Structure of **89-non-RAHB – 94-non-RAHB** compounds. Taken from Ref.50.


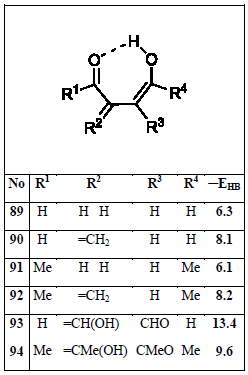


**Table S7**. Structure of **95-non-RAHB – 102-non-RAHB** compounds. Taken from Ref.50.


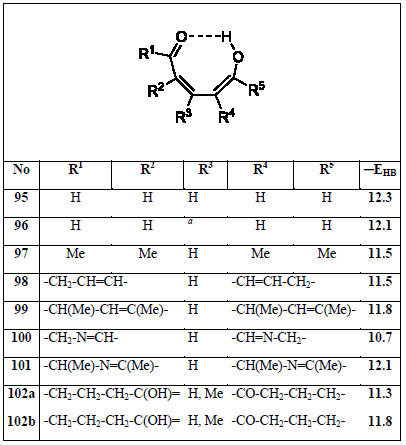


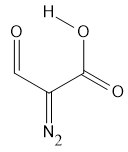
‒E_HB_ = 8.75 kcal/mol

Compound **103**

**Table S8**. The values of the spectroscopic, structural and QTAIM-based descriptors of hydrogen bond for compounds **1** – **103**. The ν_O‒H_ and ν_C=O_ vibration frequencies in cm^‒1^; the δ_OH_ chemical shifts in ppm; the *r*_O∙∙∙H_, *r*_O···O_ and *l*_O‒H_ distances in Å; the φ_HB_ hydrogen bond angles in degree; the ρ_BCP_ electron density at the hydrogen bond critical point and its Laplacian in a.u.; the V_BCP_ potential energy density of electrons in a.u.; the electron density at the ring critical point in a.u.

| **No comp** | **‒E_HB_** | descriptors | | | | | | | | | | |
| --- | --- | --- | --- | --- | --- | --- | --- | --- | --- | --- | --- | --- |
|  |  | spectroscopic | | | structural | | | | QTAIM-based | | | |
|  |  | ν_O‒H_ | ν_C=O_ | δ_OH_ | *r*_O∙∙∙H_ | φ_HB_ | *r*_O···O_ | *l*_O‒H_ | ρ_BCP_ | ∇^2^ρ | V_BCP_ | ρ_RCP_ |
| **1** | **2.04** | 3796 | 1787 | 1.99 | 2.2198 | 126.5 | 2.8975 | 0.9622 | 0.0152 | 0.0623 | ‒0.0121 | 0.0129 |
| **2a** | **2.47** | 3783 | 1785 | 2.73 | 2.1585 | 129.1 | 2.8658 | 0.9635 | 0.0170 | 0.0696 | ‒0.0145 | 0.0134 |
| **2b** | **1.41** | 3774 | 1786 | 2.44 | 2.1323 | 131.2 | 2.8610 | 0.9640 | 0.0178 | 0.0726 | ‒0.0145 | 0.0133 |
| **3** | **1.94** | 3762 | 1785 | 3.07 | 2.0915 | 133.0 | 2.8385 | 0.9650 | 0.0138 | 0.0678 | ‒0.0159 | 0.0119 |
| **4** | **2.75** | 3781 | 1765 | 2.55 | 2.1582 | 128.0 | 2.8532 | 0.9628 | 0.0172 | 0.0704 | ‒0.0140 | 0.0138 |
| **5a** | **3.21** | 3759 | 1764 | 3.43 | 2.0917 | 131.1 | 2.8204 | 0.9643 | 0.0194 | 0.0793 | ‒0.0160 | 0.0143 |
| **5b** | **2.10** | 3751 | 1763 | 3.25 | 2.0549 | 134.2 | 2.8139 | 0.9651 | 0.0208 | 0.0840 | ‒0.0174 | 0.0141 |
| **6a** | **3.26** | 3760 | 1764 | 3.40 | 2.0854 | 131.3 | 2.8164 | 0.9644 | 0.0198 | 0.0811 | ‒0.0163 | 0.0145 |
| **6b** | **3.34** | 3745 | 1757 | 3.40 | 2.0676 | 133.7 | 2.8267 | 0.9709 | 0.0195 | 0.0794 | ‒0.0161 | 0.0143 |
| **6c** | **3.11** | 3759 | 1764 | 3.47 | 2.0805 | 131.5 | 2.8137 | 0.9645 | 0.0198 | 0.0811 | ‒0.0165 | 0.0145 |
| **6d** | **2.13** | 3741 | 1762 | 2.95 | 2.0360 | 135.5 | 2.8078 | 0.9658 | 0.0215 | 0.0867 | ‒0.0182 | 0.0143 |
| **6e** | **2.19** | 3749 | 1763 | 3.40 | 2.0788 | 133.8 | 2.8339 | 0.9652 | 0.0198 | 0.0798 | ‒0.0182 | 0.0138 |
| **6f** | **2.25** | 3716 | 1761 | 4.64 | 1.9206 | 143.2 | 2.7564 | 0.9667 | 0.0271 | 0.1054 | ‒0.0239 | 0.0151 |
| **7** | **2.59** | 3691 | 1760 | 4.26 | 1.9705 | 138.4 | 2.7707 | 0.9687 | 0.0245 | 0.0970 | ‒0.0212 | 0.0150 |
| **8a** | **2.66** | 3733 | 1761 | 3.92 | 2.0018 | 136.8 | 2.7860 | 0.9665 | 0.0249 | 0.0740 | ‒0.0197 | 0.0126 |
| **8b** | **2.60** | 3733 | 1762 | 3.92 | 2.0478 | 135.4 | 2.8186 | 0.9664 | 0.0210 | 0.0843 | ‒0.0177 | 0.0140 |
| **9a** | **2.81** | 3785 | 1724 | 2.65 | 2.1622 | 126.8 | 2.8454 | 0.9626 | 0.0171 | 0.0707 | ‒0.0140 | 0.0141 |
| **9b** | **2.60** | 3777 | 1783 | 3.18 | 2.1337 | 130.5 | 2.8561 | 0.9643 | 0.0177 | 0.0725 | ‒0.0145 | 0.0137 |
| **10a** | **3.03** | 3712 | 1717 | 5.12 | 2.0639 | 139.8 | 2.8176 | 0.9679 | 0.0199 | 0.0776 | ‒0.0162 | 0.0147 |
| **10b** | **3.41** | 3758 | 1720 | 4.01 | 2.0779 | 131.0 | 2.8067 | 0.9650 | 0.0200 | 0.0818 | ‒0.0166 | 0.0148 |
| **11** | **3.21** | 3768 | 1723 | 3.51 | 2.1029 | 129.5 | 2.8160 | 0.9640 | 0.0191 | 0.0786 | ‒0.0158 | 0.0146 |
| **12** | **2.63** | 3734 | 1762 | 3.91 | 2.0161 | 136.0 | 2.7932 | 0.9663 | 0.0190 | 0.0776 | ‒0.0191 | 0.0124 |
| **13a** | **1.93** | 3807 | 1790 | 0.88 | 2.3485 | 120.4 | 2.9547 | 0.9623 | 0.0124 | 0.0515 | ‒0.0100 | 0.0120 |
| **13b** | **2.08** | 3780 | 1786 | 2.25 | 2.1677 | 128.1 | 2.8640 | 0.9629 | 0.0168 | 0.0689 | ‒0.0135 | 0.0133 |
| **13c** | **2.14** | 3770 | 1794 | 2.46 | 2.1425 | 130.3 | 2.8614 | 0.9636 | 0.0176 | 0.0712 | ‒0.0142 | 0.0132 |
| **14a** | **2.34** | 3787 | 1792 | 2.47 | 2.1904 | 127.7 | 2.8819 | 0.9626 | 0.0167 | 0.0660 | ‒0.0130 | 0.0134 |
| **14b** | **2.54** | 3783 | 1826 | 2.57 | 2.1945 | 126.8 | 2.8768 | 0.9630 | 0.0161 | 0.0660 | ‒0.0130 | 0.0136 |
| **15** | **3.69** | 3739 | 1739 | 3.53 | 2.0751 | 131.9 | 2.8127 | 0.9649 | 0.0202 | 0.0817 | ‒0.0168 | 0.0147 |
| **16** | **2.54** | 3782 | 1757 | 2.51 | 2.1682 | 127.5 | 2.8579 | 0.9626 | 0.0168 | 0.0693 | ‒0.0137 | 0.0137 |
| **17a** | **2.72** | 3770 | 1789 | 3.29 | 2.1244 | 130.6 | 2.8477 | 0.9639 | 0.0181 | 0.0668 | ‒0.0148 | 0.0139 |
| **17b** | **2.96** | 3765 | 1824 | 3.40 | 2.1280 | 129.7 | 2.8428 | 0.9643 | 0.0181 | 0.0743 | ‒0.0148 | 0.0142 |
| **18** | **4.18** | 3706 | 1736 | 4.48 | 1.9987 | 136.0 | 2.7764 | 0.9670 | 0.0233 | 0.0933 | ‒0.0199 | 0.0151 |
| **19a** | **3.11** | 3759 | 1765 | 3.57 | 2.0925 | 132.2 | 2.8320 | 0.9645 | 0.0193 | 0.0789 | ‒0.0159 | 0.0142 |
| **19b** | **3.40** | 3763 | 1786 | 3.39 | 2.1035 | 129.7 | 2.8190 | 0.9646 | 0.0192 | 0.0785 | ‒0.0158 | 0.0148 |
| **20** | **5.79** | 3492 | 1748 | 10.87 | 1.7784 | 149.7 | 2.6684 | 0.9775 | 0.0364 | 0.1318 | ‒0.0341 | 0.0178 |
| **21** | **5.53** | 3503 | 1837 | 10.51 | 1.7669 | 151.2 | 2.6651 | 0.9774 | 0.0303 | 0.0868 | ‒0.0351 | 0.0191 |
| **22** | **6.40** | 3459 | 1754 | 10.81 | 1.7549 | 149.8 | 2.6477 | 0.9792 | 0.0386 | 0.1366 | ‒0.0366 | 0.0178 |
| **23** | **3.45** | 3604 | 1874 | 9.91 | 1.8321 | 149.1 | 2.7125 | 0.9724 | 0.0318 | 0.0676 | ‒0.0292 | 0.0169 |
| **24a** | **5.21** | 3519 | 1840 | 10.67 | 1.7850 | 149.6 | 2.6729 | 0.9763 | 0.0357 | 0.1306 | ‒0.0334 | 0.0175 |
| **24b** | **5.94** | 3469 | 1842 | 11.02 | 1.7551 | 150.2 | 2.6502 | 0.9793 | 0.0385 | 0.1367 | ‒0.0363 | 0.0181 |
| **25** | **2.79** | 3664 | 1777 | 4.96 | 1.9830 | 137.2 | 2.7738 | 0.9692 | 0.0240 | 0.0950 | ‒0.0206 | 0.0157 |
| **26** | **3.32** | 3748 | 1720 | 3.18 | 2.0768 | 131.8 | 2.8126 | 0.9644 | 0.0201 | 0.0815 | ‒0.0166 | 0.0144 |
| **27** | **2.24** | 3777 | 1782 | 3.04 | 2.1335 | 129.0 | 2.8404 | 0.9637 | 0.0178 | 0.0737 | ‒0.0146 | 0.0139 |
| **28** | **5.59** | 3601 | 1719 | 8.30 | 1.9557 | 144.3 | 2.8029 | 0.9719 | 0.0245 | 0.0937 | ‒0.0210 | 0.0144 |
| **29** | **6.83** | 3570 | 1708 | 8.73 | 1.9328 | 144.9 | 2.7863 | 0.9736 | 0.0259 | 0.0975 | ‒0.0224 | 0.0148 |
| **30a** | **2.40** | 3773 | 1778 | 2.85 | 2.1232 | 129.2 | 2.8330 | 0.9640 | 0.0182 | 0.0753 | ‒0.0150 | 0.0140 |
| **30b** | **2.09** | 3763 | 1775 | 3.47 | 2.0188 | 132.0 | 2.7589 | 0.9645 | 0.0224 | 0.0929 | ‒0.0192 | 0.0152 |
| **31a** | **2.63** | 3792 | 1789 | 2.38 | 2.2077 | 126.8 | 2.8901 | 0.9637 | 0.0155 | 0.0635 | ‒0.0124 | 0.0130 |
| **31b** | **1.80** | 3774 | 1788 | 2.27 | 2.1571 | 127.4 | 2.8477 | 0.9644 | 0.0171 | 0.0708 | ‒0.0139 | 0.0137 |
| **32a** | **3.56** | 3700 | 1703 | 4.13 | 1.9889 | 140.6 | 2.8047 | 0.9679 | 0.0233 | 0.0904 | ‒0.0195 | 0.0153 |
| **32b** | **3.15** | 3779 | 1758 | 2.41 | 2.1594 | 127.6 | 2.8508 | 0.9629 | 0.0172 | 0.0706 | ‒0.0140 | 0.0140 |
| **33a** | **5.00** | 3526 | 1763 | 10.55 | 1.8001 | 148.6 | 2.6820 | 0.9762 | 0.0347 | 0.1273 | ‒0.0324 | 0.0177 |
| **33b** | **5.31** | 3479 | 1784 | 10.88 | 1.8041 | 146.4 | 2.6743 | 0.9781 | 0.0347 | 0.1260 | ‒0.0322 | 0.0183 |
| **33c** | **5.93** | 3423 | 1782 | 11.59 | 1.7469 | 149.2 | 2.6373 | 0.9804 | 0.0394 | 0.1385 | ‒0.0376 | 0.0184 |
| **33d** | **4.86** | 3547 | 1763 | 10.16 | 1.8321 | 146.8 | 2.7016 | 0.9752 | 0.0323 | 0.1207 | ‒0.0296 | 0.0174 |
| **33e** | **4.46** | 3503 | 1749 | 10.84 | 1.7897 | 147.0 | 2.6619 | 0.9761 | 0.0356 | 0.1310 | ‒0.0334 | 0.0181 |
| **34** | **12.48** | 3078 | 1632 | 14.22 | 1.6100 | 154.4 | 2.5489 | 1.0002 | 0.0553 | 0.1625 | ‒0.0563 | 0.0202 |
| **35** | **11.15** | 3129 | 1646 | 14.10 | 1.6274 | 153.6 | 2.5582 | 0.9962 | 0.0528 | 0.1608 | ‒0.0533 | 0.0199 |
| **36** | **9.35** | 3089 | 1718 | 14.19 | 1.6192 | 151.6 | 2.5367 | 0.9926 | 0.0536 | 0.1662 | ‒0.0549 | 0.0203 |
| **37a** | **9.28** | 3318 | 1699 | 12.08 | 1.7338 | 151.3 | 2.6425 | 0.9869 | 0.0407 | 0.1372 | ‒0.0387 | 0.0184 |
| **37b** | **6.92** | 3421 | 1720 | 11.21 | 1.7455 | 152.0 | 2.6513 | 0.9802 | 0.0394 | 0.1376 | ‒0.0377 | 0.0180 |
| **38** | **2.41** | 3715 | 1780 | 4.17 | 2.0581 | 135.5 | 2.8300 | 0.9673 | 0.0204 | 0.0819 | ‒0.0170 | 0.0142 |
| **39** | **3.30** | 3748 | 1756 | 3.55 | 2.0816 | 131.4 | 2.8138 | 0.9646 | 0.0198 | 0.0811 | ‒0.0165 | 0.0144 |
| **40** | **2.82** | 3789 | 1715 | 2.11 | 2.2132 | 125.9 | 2.8855 | 0.9629 | 0.0155 | 0.0634 | ‒0.0124 | 0.0130 |
| **41a** | **2.76** | 3790 | 1708 | 2.09 | 2.2212 | 125.6 | 2.8901 | 0.9628 | 0.0152 | 0.0625 | ‒0.0122 | 0.0129 |
| **41b** | **3.00** | 3799 | 1715 | 1.88 | 2.2522 | 123.7 | 2.8994 | 0.9627 | 0.0145 | 0.0599 | ‒0.0117 | 0.0131 |
| **42** | **6.43** | 3565 | 1747 | 9.17 | 2.0065 | 148.2 | 2.8824 | 0.9765 | 0.0216 | 0.0821 | ‒0.0180 | 0.0142 |
| **43** | **13.71** | 2880 | 1625 | 15.82 | 1.5474 | 149.2 | 2.4574 | 1.0003 | 0.0651 | 0.1844 | ‒0.0712 | 0.0223 |
| **44a** | **2.66** | 3787 | 1791 | 0.50 | 2.1957 | 129.9 | 2.9082 | 0.9626 | 0.0151 | 0.0613 | ‒0.0119 | 0.0094 |
| **44b** | **3.32** | 3710 | 1789 | 3.81 | 1.8980 | 157.7 | 2.8151 | 0.9654 | 0.0262 | 0.1006 | ‒0.0224 | 0.0086 |
| **45a** | **3.11** | 3706 | 1789 | 4.30 | 1.8837 | 160.4 | 2.8125 | 0.9661 | 0.0291 | 0.1007 | ‒0.0233 | 0.0069 |
| **45b** | **3.27** | 3696 | 1788 | 3.90 | 1.8965 | 156.2 | 2.8126 | 0.9665 | 0.0265 | 0.1012 | ‒0.0227 | 0.0086 |
| **46** | **3.14** | 3693 | 1788 | 4.34 | 1.8782 | 159.3 | 2.8038 | 0.9672 | 0.0275 | 0.1041 | ‒0.0239 | 0.0087 |
| **47a** | **4.59** | 3613 | 1768 | 4.06 | 1.8983 | 157.2 | 2.8182 | 0.9707 | 0.0264 | 0.0976 | ‒0.0228 | 0.0106 |
| **47b** | **4.12** | 3735 | 1742 | 3.48 | 1.9983 | 139.5 | 2.8023 | 0.9644 | 0.0224 | 0.0891 | ‒0.0187 | 0.0103 |
| **48** | **3.95** | 3688 | 1768 | 4.21 | 1.8639 | 159.4 | 2.7895 | 0.9665 | 0.0284 | 0.1073 | ‒0.0247 | 0.0090 |
| **49a** | **3.75** | 3681 | 1767 | 4.76 | 1.8491 | 162.6 | 2.7872 | 0.9673 | 0.0293 | 0.1091 | ‒0.0259 | 0.0093 |
| **50** | **3.78** | 3671 | 1766 | 4.78 | 1.8506 | 159.3 | 2.7772 | 0.9681 | 0.0300 | 0.1099 | ‒0.0259 | 0.0089 |
| **51** | **4.73** | 3734 | 1722 | 4.04 | 1.9940 | 137.8 | 2.7858 | 0.9651 | 0.0228 | 0.0915 | ‒0.0192 | 0.0132 |
| **52** | **5.90** | 3525 | 1756 | 9.82 | 1.8155 | 157.0 | 2.7394 | 0.9748 | 0.0319 | 0.1153 | ‒0.0285 | 0.0107 |
| **53a** | **3.34** | 3720 | 1789 | 3.89 | 1.8933 | 156.9 | 2.8066 | 0.9650 | 0.0263 | 0.1026 | ‒0.0102 | 0.0087 |
| **53b** | **3.39** | 3717 | 1825 | 4.00 | 1.8982 | 155.1 | 2.8033 | 0.9652 | 0.0260 | 0.1020 | ‒0.0077 | 0.0090 |
| **53c** | **2.73** | 3796 | 1830 | 5.59 | 2.3094 | 124.0 | 2.9570 | 0.9625 | 0.0132 | 0.0527 | ‒0.0102 | 0.0108 |
| **54a** | **3.89** | 3703 | 1766 | 4.20 | 1.8696 | 158.1 | 2.7894 | 0.9661 | 0.0279 | 0.1075 | ‒0.0243 | 0.0087 |
| **54b** | **3.74** | 3713 | 1788 | 4.04 | 1.8895 | 154.7 | 2.7930 | 0.9655 | 0.0265 | 0.1038 | ‒0.0229 | 0.0095 |
| **55** | **4.12** | 3682 | 1760 | 4.34 | 1.8553 | 160.2 | 2.7841 | 0.9669 | 0.0290 | 0.1091 | ‒0.0254 | 0.0090 |
| **56a** | **5.13** | 3571 | 1775 | 9.49 | 1.8419 | 156.5 | 2.7616 | 0.9728 | 0.0298 | 0.1106 | ‒0.0263 | 0.0104 |
| **56b** | **5.38** | 3560 | 1830 | 9.58 | 1.8413 | 155.7 | 2.7577 | 0.9734 | 0.0298 | 0.1105 | ‒0.0264 | 0.0106 |
| **57a** | **5.45** | 3557 | 1744 | 9.63 | 1.8443 | 157.9 | 2.7704 | 0.9735 | 0.0296 | 0.1091 | ‒0.0260 | 0.0105 |
| **57b** | **5.82** | 3474 | 1771 | 10.61 | 1.7668 | 169.0 | 2.7325 | 0.9774 | 0.0356 | 0.1240 | ‒0.0322 | 0.0108 |
| **58** | **5.91** | 3351 | 1761 | 11.57 | 1.7329 | 160.2 | 2.6764 | 0.9809 | 0.0391 | 0.1334 | ‒0.0365 | 0.0114 |
| **59** | **6.31** | 3438 | 1760 | 10.18 | 1.9792 | 121.2 | 2.7021 | 0.9793 | 0.0367 | 0.1279 | ‒0.0336 | 0.0103 |
| **60** | **3.08** | 3769 | 1787 | 1.63 | 2.1839 | 159.1 | 3.1032 | 0.9638 | 0.0130 | 0.0408 | ‒0.0099 | 0.0060 |
| **61** | **3.75** | 3722 | 1767 | 3.14 | 2.0861 | 166.1 | 3.0314 | 0.9648 | 0.0146 | 0.0484 | ‒0.0125 | 0.0131 |
| **62** | **4.14** | 3674 | 1768 | 3.97 | 1.9012 | 167.7 | 2.8545 | 0.9681 | 0.0251 | 0.0947 | ‒0.0210 | 0.0052 |
| **63a** | **5.88** | 3585 | 1775 | 9.72 | 1.8802 | 173.7 | 2.8489 | 0.9726 | 0.0254 | 0.0963 | ‒0.0215 | 0.0083 |
| **63b** | **6.05** | 3583 | 1808 | 9.89 | 1.8758 | 173.0 | 2.8440 | 0.9730 | 0.0256 | 0.0972 | ‒0.0217 | 0.0085 |
| **64a** | **6.20** | 3542 | 1769 | 10.10 | 1.8392 | 173.6 | 2.8103 | 0.9750 | 0.0280 | 0.1045 | ‒0.0243 | 0.0086 |
| **64b** | **6.50** | 3556 | 1802 | 9.88 | 1.8565 | 173.8 | 2.8278 | 0.9750 | 0.0271 | 0.1082 | ‒0.0231 | 0.0072 |
| **64c** | **6.40** | 3534 | 1803 | 10.24 | 1.8066 | 172.6 | 2.7762 | 0.9749 | 0.0304 | 0.1124 | ‒0.0267 | 0.0074 |
| **65** | **4.44** | 3561 | 1789 | 8.96 | 1.8896 | 154.6 | 2.7988 | 0.9718 | 0.0261 | 0.0997 | ‒0.0225 | 0.0091 |
| **66** | **4.03** | 3616 | 1784 | 8.36 | 1.9376 | 150.5 | 2.8232 | 0.9710 | 0.0240 | 0.0924 | ‒0.0204 | 0.0084 |
| **67a** | **6.01** | 3543 | 1765 | 9.95 | 1.8172 | 177.2 | 2.7913 | 0.9749 | 0.0301 | 0.1091 | ‒0.0264 | 0.0067 |
| **67b** | **6.23** | 3530 | 1816 | 10.22 | 1.8070 | 177.4 | 2.7819 | 0.9756 | 0.0308 | 0.1112 | ‒0.0272 | 0.0087 |
| **68a** | **3.71** | 3541 | 1768 | 10.57 | 1.8437 | 147.4 | 2.7123 | 0.9708 | 0.0292 | 0.1131 | ‒0.0263 | 0.0099 |
| **68b** | **4.26** | 3509 | 1808 | 10.97 | 1.7948 | 151.1 | 2.6882 | 0.9731 | 0.0324 | 0.1220 | ‒0.0297 | 0.0101 |
| **69a** | **5.31** | 3632 | 1771 | 8.69 | 1.9523 | 169.5 | 2.9118 | 0.9704 | 0.0202 | 0.0816 | ‒0.0165 | 0.0065 |
| **69b** | **5.30** | 3637 | 1804 | 8.74 | 1.9578 | 170.4 | 2.9190 | 0.9703 | 0.0197 | 0.0802 | ‒0.0161 | 0.0064 |
| **70** | **2.03** | 3788 | 1755 | 2.61 | 2.1947 | 126.7 | 2.8747 | 0.9622 | 0.0162 | 0.0657 | ‒0.0129 | 0.0134 |
| **71a** | **2.32** | 3790 | 1744 | 2.43 | 2.1796 | 126.8 | 2.8610 | 0.9623 | 0.0165 | 0.0680 | ‒0.0134 | 0.0135 |
| **71b** | **2.16** | 3797 | 1746 | 2.26 | 2.1967 | 126.2 | 2.8715 | 0.9620 | 0.0160 | 0.0659 | ‒0.0130 | 0.0134 |
| **72a** | **2.61** | 3781 | 1743 | 3.08 | 2.1321 | 129.0 | 2.8385 | 0.9634 | 0.0180 | 0.0741 | ‒0.0148 | 0.0139 |
| **72b** | **2.79** | 3775 | 1742 | 3.20 | 2.1108 | 130.0 | 2.8280 | 0.9637 | 0.0186 | 0.0768 | ‒0.0154 | 0.0139 |
| **73a** | **2.04** | 3765 | 1740 | 3.77 | 2.0443 | 133.9 | 2.8009 | 0.9651 | 0.0205 | 0.0865 | ‒0.0180 | 0.0134 |
| **73b** | **2.17** | 3753 | 1739 | 3.72 | 2.0341 | 134.8 | 2.7991 | 0.9654 | 0.0216 | 0.0875 | ‒0.0183 | 0.0142 |
| **74** | **2.57** | 3797 | 1706 | 2.13 | 2.2240 | 125.1 | 2.8867 | 0.9626 | 0.0152 | 0.0626 | ‒0.0122 | 0.0129 |
| **75** | **2.60** | 3804 | 1682 | 1.76 | 2.2550 | 122.8 | 2.8922 | 0.9624 | 0.0145 | 0.0602 | ‒0.0117 | 0.0131 |
| **76** | **3.22** | 3800 | 1754 | 0.87 | 2.3189 | 122.8 | 2.9531 | 0.9622 | 0.0130 | 0.0521 | ‒0.0101 | 0.0109 |
| **77** | **3.33** | 3788 | 1749 | 0.28 | 2.2613 | 125.4 | 2.9276 | 0.9640 | 0.0143 | 0.0579 | ‒0.0112 | 0.0116 |
| **78** | **2.87** | 3747 | 1749 | 2.57 | 2.2123 | 158.7 | 3.1296 | 0.9636 | 0.0125 | 0.0492 | ‒0.0094 | 0.0138 |
| **79** | **6.56** | 3325 | 1739 | 12.75 | 1.6509 | 172.3 | 2.6280 | 0.9826 | 0.0473 | 0.1541 | ‒0.0459 | 0.0097 |
| **80** | **5.77** | 3334 | 1727 | 10.66 | 1.6682 | 170.6 | 2.6399 | 0.9800 | 0.0456 | 0.1491 | ‒0.0437 | 0.0094 |
| **81a** | **5.25** | 3422 | 1741 | 10.40 | 1.6764 | 170.1 | 2.6440 | 0.9769 | 0.0440 | 0.1492 | ‒0.0422 | 0.0094 |
| **81b** | **5.66** | 3383 | 1768 | 10.74 | 1.6615 | 169.9 | 2.6307 | 0.9788 | 0.0458 | 0.1526 | ‒0.0442 | 0.0095 |
| **82a** | **6.41** | 3372 | 1790 | 12.39 | 1.6538 | 171.9 | 2.6286 | 0.9809 | 0.0464 | 0.1547 | ‒0.0450 | 0.0097 |
| **82b** | **7.01** | 3323 | 1797 | 12.77 | 1.6357 | 171.8 | 2.6127 | 0.9834 | 0.0487 | 0.1588 | ‒0.0477 | 0.0099 |
| **83a** | **9.22** | 3085 | 1709 | 13.78 | 1.5645 | 168.1 | 2.5447 | 0.9934 | 0.0587 | 0.1737 | ‒0.0606 | 0.0099 |
| **84a** | **9.20** | 3150 | 1732 | 12.60 | 1.6053 | 167.7 | 2.5819 | 0.9907 | 0.0528 | 0.1626 | ‒0.0525 | 0.0096 |
| **84b** | **10.07** | 3083 | 1753 | 13.14 | 1.5865 | 167.9 | 2.5669 | 0.9940 | 0.0555 | 0.1661 | ‒0.0559 | 0.0098 |
| **85a** | **6.32** | 3301 | 1715 | 12.20 | 1.6212 | 169.4 | 2.5928 | 0.9821 | 0.0506 | 0.1631 | ‒0.0504 | 0.0098 |
| **86a** | **9.61** | 3158 | 1694 | 13.93 | 1.5896 | 173.5 | 2.5776 | 0.9920 | 0.0553 | 0.1672 | ‒0.0558 | 0.0101 |
| **86b** | **9.88** | 3131 | 1696 | 14.11 | 1.5787 | 173.2 | 2.5675 | 0.9931 | 0.0568 | 0.1716 | ‒0.0578 | 0.0102 |
| **87** | **9.02** | 3196 | 1712 | 13.60 | 1.5981 | 173.1 | 2.5836 | 0.9900 | 0.0540 | 0.1661 | ‒0.0541 | 0.0101 |
| **88a** | **4.84** | 3492 | 1692 | 10.31 | 1.7767 | 155.3 | 2.6915 | 0.9732 | 0.0344 | 0.1263 | ‒0.0317 | 0.0103 |
| **88b** | **5.36** | 3468 | 1717 | 10.81 | 1.7350 | 157.6 | 2.6623 | 0.9749 | 0.0379 | 0.1359 | ‒0.0357 | 0.0101 |
| **89** | **6.32** | 3507 | 1780 | 9.17 | 1.7243 | 164.6 | 2.6773 | 0.9755 | 0.0394 | 0.1377 | ‒0.0366 | 0.0087 |
| **90** | **8.13** | 3376 | 1732 | 10.81 | 1.6842 | 165.1 | 2.6439 | 0.9807 | 0.0437 | 0.1465 | ‒0.0415 | 0.0090 |
| **91** | **6.05** | 3542 | 1744 | 8.34 | 1.8639 | 155.1 | 2.7775 | 0.9738 | 0.0287 | 0.1044 | ‒0.0251 | 0.0109 |
| **92** | **8.19** | 3338 | 1706 | 11.86 | 1.6327 | 166.4 | 2.5976 | 0.9821 | 0.0495 | 0.1617 | ‒0.0488 | 0.0097 |
| **93** | **13.36** | 2647 | 1696 | 16.49 | 1.4887 | 168.8 | 2.4921 | 1.0149 | 0.0734 | 0.1748 | ‒0.0808 | 0.0101 |
| **94** | **9.58** | 3068 | 1647 | 12.55 | 1.6390 | 161.6 | 2.5978 | 0.9906 | 0.0499 | 0.1537 | ‒0.0489 | 0.0127 |
| **95** | **12.32** | 2727 | 1696 | 14.03 | 1.5511 | 171.2 | 2.5418 | 0.9980 | 0.0604 | 0.1710 | ‒0.0619 | 0.0063 |
| **96** | **12.07** | 2988 | 1703 | 8.29 | 1.5896 | 163.9 | 2.5538 | 0.9883 | 0.0548 | 0.1696 | ‒0.0555 | 0.0082 |
| **97** | **11.46** | 2805 | 1661 | 9.57 | 1.5709 | 165.8 | 2.5422 | 0.9900 | 0.0579 | 0.1735 | ‒0.0595 | 0.0091 |
| **98** | **11.51** | 2833 | 1700 | 14.22 | 1.5571 | 173.9 | 2.5509 | 0.9972 | 0.0593 | 0.1688 | ‒0.0604 | 0.0058 |
| **99** | **11.83** | 2782 | 1693 | 13.63 | 1.5518 | 173.5 | 2.5466 | 0.9987 | 0.0602 | 0.1692 | ‒0.0616 | 0.0058 |
| **100** | **10.68** | 2830 | 1722 | 15.84 | 1.5829 | 179.3 | 2.5799 | 0.9971 | 0.0555 | 0.1614 | ‒0.0550 | 0.0045 |
| **101** | **12.08** | 2770 | 1712 | 15.38 | 1.5619 | 174.5 | 2.5584 | 0.9992 | 0.0586 | 0.1655 | ‒0.0592 | 0.0054 |
| **102a** | **11.29** | 3070 | 1647 | 12.93 | 1.6537 | 167.4 | 2.6346 | 0.9960 | 0.0464 | 0.1421 | ‒0.0439 | 0.0140 |
| **102b** | **11.76** | 2959 | 1647 | 13.40 | 1.6004 | 168.0 | 2.5861 | 0.9993 | 0.0533 | 0.1558 | ‒0.0525 | 0.0106 |
| **103** | **8.75** | 3400 | 1690 | 10.53 | 1.7857 | 148.7 | 2.6775 | 0.9858 | 0.0361 | 0.1266 | ‒0.0334 | 0.0169 |

**Table S9.** The values of the NBO-based descriptors for compounds **1**–**103**. LP_1_ → σ*(O–H) – the energy of charge transfer from the first lone pair of oxygen to the antibonding orbital of the O–H bond due to the O–H···O=C hydrogen bond (in kcal/mol); LP_2_(O) → σ*(O–H) – the energy of charge transfer from the second lone pair of oxygen to the antibonding orbital of the O–H bond due to the O–H···O=C hydrogen bond (in kcal/mol); Σ(σ→ σ*) – the sum of charge transfer energy from both lone pair of oxygen (in kcal/mol); n(LP_1_) and n(LP_2_) are the orbital occupancies of the first and second oxygen lone pairs; [n(LP_1_) + n(LP_2_)] – the sum of the lone pairs occupancies; n[σ*(O‒H)] – the electron occupancy of the antibonding σ*(O–H) orbital (in *e*); W(O···H), W(O–H) and W(C=O) – the Wiberg indices for the O···H hydrogen bond, O–H and C=O covalent bonds, respectively; P(O–H), σ-P(C=O) and π-P(C=O) – the polarization of the O–H bond, σ- and π-bond of the C=O group, respectively (in %).

| **No compounds** | ‒*E*_HB_(MTA) | LP_1_(O)→ σ*(O–H) | LP_2_(O)→ σ*(O–H) | Σ(σ→σ*) | n(LP_1_) | n(LP_2_) | [n(LP_1_)+ n(LP_2_)] | n[σ*(O‒H)] | W(O···H) | W(O–H) | W(C=O) | P(O–H) | σ-P(C=O) | π-P(C=O) |
| --- | --- | --- | --- | --- | --- | --- | --- | --- | --- | --- | --- | --- | --- | --- |
| **1** | **2.04** | – | 1.12 | 1.12 | 1.98554 | 1.91758 | 3.90312 | 0.00764 | 0.0046 | 0.7514 | 1.7853 | 74.46 | 66.01 | 71.00 |
| **2a** | **2.47** | – | 1.59 | 1.59 | 1.98533 | 1.91738 | 3.90271 | 0.00789 | 0.0061 | 0.7452 | 1.7826 | 74.71 | 66.01 | 71.09 |
| **2b** | **1.41** | – | 2.02 | 2.02 | 1.98490 | 1.91683 | 3.90173 | 0.01025 | 0.0070 | 0.7462 | 1.7798 | 74.58 | 66.06 | 71.20 |
| **3** | **1.94** | 0.57 | 2.52 | 3.09 | 1.98466 | 1.91629 | 3.90095 | 0.01057 | 0.0085 | 0.7401 | 1.7774 | 74.82 | 66.06 | 71.29 |
| **4** | **2.75** | – | 1.57 | 1.57 | 1.97889 | 1.92335 | 3.90224 | 0.00863 | 0.0059 | 0.7469 | 1.7330 | 74.65 | 66.42 | 71.91 |
| **5a** | **3.21** | – | 2.35 | 2.35 | 1.97865 | 1.92279 | 3.90144 | 0.00942 | 0.0081 | 0.7391 | 1.7299 | 74.95 | 66.42 | 72.03 |
| **5b** | **2.10** | 0.66 | 3.35 | 4.01 | 1.97794 | 1.92168 | 3.89962 | 0.01222 | 0.0100 | 0.7379 | 1.7258 | 74.93 | 66.51 | 72.19 |
| **6а** | **3.26** | – | 2.43 | 2.43 | 1.97860 | 1.92279 | 3.90139 | 0.00972 | 0.0084 | 0.7379 | 1.7290 | 75.01 | 66.43 | 72.06 |
| **6b** | **3.34** | – | 2.46 | 2.46 | 1.97858 | 1.92248 | 3.90106 | 0.00967 | 0.0083 | 0.7379 | 1.7286 | 75.01 | 66.44 | 72.09 |
| **6c** | **3.11** | – | 2.45 | 2.45 | 1.97856 | 1.92274 | 3.9013 | 0.00979 | 0.0085 | 0.7387 | 1.7292 | 74.97 | 66.44 | 72.05 |
| **6d** | **2.13** | 0.75 | 3.66 | 4.41 | 1.97771 | 1.92127 | 3.89898 | 0.01295 | 0.0111 | 0.7357 | 1.7243 | 75.00 | 66.52 | 72.25 |
| **6e** | **2.19** | 0.57 | 2.98 | 3.55 | 1.97797 | 1.92200 | 3.89997 | 0.01165 | 0.0092 | 0.7392 | 1.7264 | 74.86 | 66.51 | 72.15 |
| **6f** | **2.25** | 1.41 | 6.81 | 8.22 | 1.97585 | 1.91730 | 3.89315 | 0.01779 | 0.0194 | 0.7204 | 1.7153 | 75.61 | 66.71 | 72.41 |
| **7** | **2.59** | 1.13 | 5.35 | 6.48 | 1.97712 | 1.91947 | 3.89659 | 0.01378 | 0.0157 | 0.7200 | 1.7228 | 75.70 | 66.53 | 72.29 |
| **8a** | **2.66** | 0.88 | 4.34 | 5.22 | 1.97752 | 1.92056 | 3.89808 | 0.01373 | 0.0129 | 0.7287 | 1.7217 | 75.28 | 66.53 | 72.33 |
| **8b** | **2.60** | 0.68 | 3.55 | 4.23 | 1.97767 | 1.92136 | 3.89903 | 0.01222 | 0.0107 | 0.7336 | 1.7243 | 75.07 | 66.53 | 72.20 |
| **9a** | **2.81** | – | 1.34 | 1.34 | 1.97967 | 1.92365 | 3.90332 | 0.00858 | 0.0057 | 0.7476 | 1.6921 | 74.60 | 66.11 | 72.38 |
| **9b** | **2.60** | – | 1.86 | 1.86 | 1.98522 | 1.91746 | 3.90268 | 0.00853 | 0.0070 | 0.7395 | 1.7804 | 74.97 | 66.02 | 71.17 |
| **10a** | **3.03** | 0.58 | 2.45 | 3.03 | 1.97774 | 1.92281 | 3.90055 | 0.01352 | 0.0122 | 0.7329 | 1.6918 | 75.08 | 66.38 | 72.36 |
| **10b** | **3.41** | 0.55 | 2.23 | 2.78 | 1.97927 | 1.92341 | 3.90268 | 0.00989 | 0.0087 | 0.7344 | 1.6857 | 75.16 | 66.11 | 72.60 |
| **11** | **3.21** | 0 | 1.89 | 1.89 | 1.9747 | 1.92347 | 3.89817 | 0.00906 | 0.0075 | 0.7407 | 1.6895 | 74.88 | 66.11 | 72.47 |
| **12** | **2.63** | 0.82 | 4.02 | 4.84 | 1.97768 | 1.92076 | 3.89844 | 0.01302 | 0.0121 | 0.7310 | 1.7233 | 75.18 | 66.51 | 72.28 |
| **13a** | **1.93** | – | 0.5 | 0.5 | 1.98642 | 1.91793 | 3.90435 | 0.00608 | 0.0026 | 0.7564 | 1.7995 | 74.35 | 66.13 | 70.56 |
| **13b** | **2.08** | – | 1.67 | 1.67 | 1.98617 | 1.91656 | 3.90273 | 0.00824 | 0.0057 | 0.7457 | 1.7981 | 74.72 | 66.11 | 70.49 |
| **13c** | **2.14** | – | 2.19 | 2.19 | 1.98605 | 1.91536 | 3.90141 | 0.00901 | 0.0070 | 0.7416 | 1.7969 | 74.89 | 66.28 | 70.62 |
| **14a** | **2.34** | – | 1.35 | 1.35 | 1.97971 | 1.89325 | 3.87296 | 0.00710 | 0.0048 | 0.7495 | 1.6744 | 74.54 | 65.87 | 75.14 |
| **14b** | **2.54** | – | 1.28 | 1.28 | 1.98060 | 1.88949 | 3.87009 | 0.00806 | 0.0051 | 0.7469 | 1.7054 | 74.67 | 65.78 | 73.79 |
| **15** | **3.69** | 0.61 | 2.81 | 3.42 | 1.97879 | 1.90562 | 3.88441 | 0.01082 | 0.0095 | 0.7361 | 1.5972 | 75.11 | 65.21 | 76.15 |
| **16** | **2.54** | – | 1.42 | 1.42 | 1.97895 | 1.92392 | 3.90287 | 0.00841 | 0.0055 | 0.7479 | 1.7251 | 74.60 | 66.47 | 72.06 |
| **17a** | **2.72** | – | 1.99 | 1.99 | 1.97948 | 1.89300 | 3.87248 | 0.00834 | 0.0066 | 0.7427 | 1.6714 | 74.82 | 65.87 | 75.24 |
| **17b** | **2.96** | – | 1.90 | 1.9 | 1.98037 | 1.88932 | 3.86969 | 0.00853 | 0.0070 | 0.7399 | 1.7024 | 74.95 | 65.78 | 73.89 |
| **18** | **4.18** | 1.01 | 4.52 | 5.53 | 1.98037 | 1.90418 | 3.88455 | 0.01287 | 0.0138 | 0.7250 | 1.5918 | 75.63 | 65.17 | 76.37 |
| **19a** | **3.11** | 0.51 | 2.37 | 2.88 | 1.97865 | 1.89290 | 3.87155 | 0.00913 | 0.0078 | 0.7396 | 1.6547 | 74.94 | 65.98 | 75.32 |
| **19b** | **3.40** | – | 2.02 | 2.02 | 1.98010 | 1.89190 | 3.872 | 0.00915 | 0.0078 | 0.7380 | 1.6851 | 75.02 | 65.79 | 74.22 |
| **20** | **5.79** | 3.42 | 13.56 | 16.98 | 1.97493 | 1.88283 | 3.85776 | 0.02873 | 0.0383 | 0.6757 | 1.6260 | 77.30 | 66.07 | 75.81 |
| **21** | **5.53** | 3.48 | 15.25 | 18.73 | 1.98084 | 1.90437 | 3.88521 | 0.03074 | 0.0414 | 0.6746 | 1.7691 | 77.31 | 66.01 | 71.61 |
| **22** | **6.40** | 3.05 | 16.25 | 19.3 | 1.97502 | 1.90677 | 3.88179 | 0.03275 | 0.0442 | 0.6698 | 1.7113 | 77.44 | 66.47 | 72.70 |
| **23** | **3.45** | 2.50 | 10.71 | 13.21 | 1.97852 | 1.86139 | 3.83991 | 0.02271 | 0.0292 | 0.6902 | 1.7724 | 76.83 | 66.20 | 73.01 |
| **24a** | **5.21** | 3.21 | 13.33 | 16.54 | 1.97591 | 1.88259 | 3.8585 | 0.02763 | 0.0367 | 0.6782 | 1.6470 | 77.23 | 65.94 | 75.62 |
| **24b** | **5.94** | 3.86 | 15.99 | 19.85 | 1.97593 | 1.88483 | 3.86076 | 0.03204 | 0.0441 | 0.6673 | 1.6718 | 77.59 | 65.99 | 74.32 |
| **25** | **2.79** | 1.11 | 4.88 | 5.99 | 1.97813 | 1.89180 | 3.86993 | 0.01461 | 0.0149 | 0.7183 | 1.6484 | 75.85 | 65.92 | 75.82 |
| **26** | **3.32** | 0.57 | 2.81 | 3.38 | 1.97912 | 1.92433 | 3.90345 | 0.01074 | 0.0092 | 0.7574 | 1.6920 | 75.03 | 65.94 | 72.21 |
| **27** | **2.24** | – | 1.70 | 1.7 | 1.98513 | 1.91765 | 3.90278 | 0.00828 | 0.0065 | 0.7424 | 1.7851 | 74.83 | 66.11 | 71.07 |
| **28** | **5.59** | 1.40 | 7.00 | 8.4 | 1.98459 | 1.91637 | 3.90096 | 0.01748 | 0.0204 | 0.6975 | 1.7008 | 76.63 | 65.39 | 72.16 |
| **29** | **6.83** | 1.55 | 8.02 | 9.57 | 1.98427 | 1.91548 | 3.89975 | 0.01923 | 0.0233 | 0.6937 | 1.6582 | 76.76 | 65.36 | 73.42 |
| **30a** | **2.40** | – | 1.81 | 1.81 | 1.98496 | 1.91783 | 3.90279 | 0.00874 | 0.0068 | 0.7427 | 1.7800 | 74.80 | 66.15 | 71.23 |
| **30b** | **2.09** | 0.85 | 2.95 | 3.8 | 1.98406 | 1.91526 | 3.89932 | 0.01136 | 0.0107 | 0.7335 | 1.7758 | 75.18 | 66.23 | 71.35 |
| **31a** | **2.63** | – | 1.26 | 1.26 | 1.98529 | 1.91503 | 3.90032 | 0.00708 | 0.0049 | 0.7455 | 1.7924 | 74.72 | 66.02 | 70.94 |
| **31b** | **1.80** | – | 1.40 | 1.4 | 1.98505 | 1.91559 | 3.90064 | 0.00822 | 0.0056 | 0.7452 | 1.7913 | 74.64 | 66.05 | 70.91 |
| **32a** | **3.56** | 0.98 | 3.39 | 4.37 | 1.97878 | 1.92329 | 3.90207 | 0.01531 | 0.0159 | 0.7238 | 1.6581 | 75.53 | 65.63 | 72.91 |
| **32b** | **3.15** | – | 1.43 | 1.43 | 1.97827 | 1.92462 | 3.90289 | 0.00865 | 0.0058 | 0.7468 | 1.7377 | 74.63 | 66.54 | 71.90 |
| **33a** | **5.00** | 2.88 | 12.31 | 15.19 | 1.97549 | 1.88305 | 3.85854 | 0.02674 | 0.0349 | 0.6818 | 1.6403 | 77.06 | 66.15 | 75.81 |
| **33b** | **5.31** | 3.03 | 11.36 | 14.39 | 1.97622 | 1.88101 | 3.85723 | 0.02806 | 0.0373 | 0.6743 | 1.6538 | 77.35 | 66.80 | 74.02 |
| **33c** | **5.93** | 4.03 | 15.85 | 19.88 | 1.97506 | 1.87731 | 3.85237 | 0.03351 | 0.0462 | 0.6638 | 1.6513 | 77.68 | 67.11 | 73.80 |
| **33d** | **4.86** | 2.42 | 10.47 | 12.89 | 1.97591 | 1.88516 | 3.86107 | 0.02385 | 0.0304 | 0.6863 | 1.6417 | 76.93 | 66.14 | 75.77 |
| **33e** | **4.46** | 3.13 | 12.02 | 15.15 | 1.97540 | 1.88336 | 3.85876 | 0.02738 | 0.0358 | 0.6785 | 1.6330 | 77.20 | 66.92 | 75.09 |
| **34** | **12.48** | 4.73 | 35.29 | 40.02 | 1.97328 | 1.89409 | 3.86737 | 0.05864 | 0.0854 | 0.6137 | 1.4942 | 79.22 | 65.17 | 77.06 |
| **35^a^** | **11.15** | – | – | – | – | – | – | – | – | – | – | – | – | – |
| **36^a^** | **9.35** | – | – | – | – | – | – | – | – | – | – | – | – | – |
| **37a** | **9.28** | 3.78 | 19.69 | 23.47 | 1.98027 | 1.89912 | 3.87939 | 0.03804 | 0.0534 | 0.6504 | 1.6503 | 78.15 | 65.34 | 73.92 |
| **37b** | **6.92** | 3.55 | 17.53 | 21.08 | 1.98024 | 1.89934 | 3.87958 | 0.03436 | 0.0470 | 0.6679 | 1.6715 | 77.51 | 65.46 | 73.17 |
| **38** | **2.41** | 0.68 | 3.36 | 4.04 | 1.97799 | 1.89228 | 3.87027 | 0.01229 | 0.0101 | 0.7273 | 1.6565 | 75.36 | 66.10 | 75.51 |
| **39** | **3.30** | 0.53 | 2.36 | 2.89 | 1.97843 | 1.92299 | 3.90142 | 0.00977 | 0.0084 | 0.7381 | 1.7195 | 74.99 | 66.52 | 72.30 |
| **40** | **2.82** | – | 1.10 | 1.1 | 1.98164 | 1.92940 | 3.91104 | 0.00747 | 0.0045 | 0.7493 | 1.7015 | 74.56 | 65.32 | 70.08 |
| **41a** | **2.76** | – | 1.06 | 1.06 | 1.98171 | 1.92965 | 3.91136 | 0.00737 | 0.0043 | 0.7492 | 1.7036 | 74.57 | 65.29 | 69.96 |
| **41b** | **3.00** | – | 0.82 | 0.82 | 1.98175 | 1.92963 | 3.91138 | 0.00686 | 0.0037 | 0.7522 | 1.7026 | 74.44 | 65.35 | 69.97 |
| **42** | **6.43** | 1.03 | 4.42 | 5.45 | 1.97811 | 1.88162 | 3.85973 | 0.03242 | 0.0428 | 0.6366 | 1.5697 | 76.64 | 65.25 | 72.73 |
| **43** | **13.71** | 5.28 | 40.29 | 50.57 | 1.97155 | 1.89270 | 3.86425 | 0.06819 | 0.0909 | 0.5983 | 1.5390 | 79.71 | 66.30 | 76.66 |
| **44a** | **2.66** | – | 0.81 | 0.81 | 1.98490 | 1.91582 | 3.90072 | 0.00854 | 0.0048 | 0.7525 | 1.7766 | 74.37 | 66.12 | 71.46 |
| **44b** | **3.32** | 2.98 | 6.16 | 9.14 | 1.98129 | 1.91342 | 3.89471 | 0.01807 | 0.0196 | 0.7230 | 1.7696 | 75.54 | 66.13 | 71.56 |
| **45a** | **3.11** | 3.05 | 6.76 | 9.81 | 1.98107 | 1.91281 | 3.89388 | 0.01839 | 0.0213 | 0.7183 | 1.7688 | 75.71 | 66.13 | 71.57 |
| **45b** | **3.27** | 3.12 | 6.43 | 9.55 | 1.98099 | 1.91341 | 3.8944 | 0.01877 | 0.0197 | 0.7220 | 1.7679 | 75.51 | 66.14 | 71.60 |
| **46** | **3.14** | 3.30 | 7.19 | 10.49 | 1.98063 | 1.91274 | 3.89337 | 0.01930 | 0.0215 | 0.7165 | 1.7666 | 75.70 | 66.15 | 71.62 |
| **47a** | **4.59** | 2.07 | 6.96 | 9.03 | 1.98208 | 1.91182 | 3.8939 | 0.02175 | 0.0253 | 0.6969 | 1.7642 | 76.51 | 66.34 | 71.75 |
| **47b** | **4.12** | 1.45 | 3.20 | 4.65 | 1.98301 | 1.91433 | 3.89734 | 0.01225 | 0.0117 | 0.7326 | 1.7224 | 75.22 | 65.70 | 71.89 |
| **48** | **3.95** | 3.09 | 7.79 | 10.88 | 1.97462 | 1.91825 | 3.89287 | 0.02023 | 0.0229 | 0.7161 | 1.7179 | 75.81 | 66.52 | 72.49 |
| **49a** | **3.75** | 3.11 | 8.59 | 11.7 | 1.97442 | 1.91732 | 3.89174 | 0.02098 | 0.0251 | 0.7107 | 1.7171 | 75.99 | 66.52 | 72.51 |
| **50** | **3.78** | 3.35 | 8.44 | 11.79 | 1.97398 | 1.91801 | 3.89199 | 0.02082 | 0.0241 | 0.7112 | 1.7153 | 75.90 | 66.56 | 72.53 |
| **51** | **4.73** | 1.46 | 3.69 | 5.15 | 1.97747 | 1.92339 | 3.90086 | 0.01238 | 0.0114 | 0.7313 | 1.6933 | 75.29 | 66.16 | 72.27 |
| **52** | **5.90** | 3.41 | 10.47 | 13.88 | 1.97385 | 1.91385 | 3.8877 | 0.02592 | 0.0331 | 0.6845 | 1.7037 | 76.97 | 66.62 | 73.02 |
| **53a** | **3.34** | 3.00 | 6.26 | 9.26 | 1.97584 | 1.89122 | 3.86706 | 0.01686 | 0.0185 | 0.7235 | 1.6595 | 75.56 | 66.95 | 75.60 |
| **53b** | **3.39** | 3.11 | 5.84 | 8.95 | 1.97662 | 1.88804 | 3.86466 | 0.01665 | 0.0184 | 0.7221 | 1.6911 | 75.64 | 65.86 | 74.23 |
| **53c** | **2.73** | – | – | – | 1.97997 | 1.88811 | 3.86808 | 0.00740 | 0.0028 | 0.7557 | 1.6967 | 74.24 | 66.19 | 74.03 |
| **54a** | **3.89** | 3.53 | 7.16 | 10.69 | 1.97444 | 1.89052 | 3.86496 | 0.01857 | 0.0210 | 0.7187 | 1.6418 | 75.74 | 66.07 | 75.73 |
| **54b** | **3.74** | 3.39 | 5.46 | 8.85 | 1.97614 | 1.89101 | 3.86715 | 0.01705 | 0.0192 | 0.7203 | 1.6728 | 75.71 | 65.95 | 74.49 |
| **55** | **4.12** | 3.37 | 8.06 | 11.43 | 1.97408 | 1.91864 | 3.89272 | 0.02102 | 0.0239 | 0.7146 | 1.7041 | 75.85 | 66.64 | 72.89 |
| **56a** | **5.13** | 3.47 | 8.28 | 11.75 | 1.97490 | 1.89051 | 3.86541 | 0.02233 | 0.0281 | 0.6915 | 1.6410 | 76.78 | 66.01 | 76.01 |
| **56b** | **5.38** | 3.66 | 8.13 | 11.79 | 1.97556 | 1.88718 | 3.86274 | 0.02291 | 0.0292 | 0.6883 | 1.6702 | 76.90 | 65.91 | 74.74 |
| **57a** | **5.45** | 3.41 | 8.32 | 11.73 | 1.97566 | 1.89289 | 3.86855 | 0.02266 | 0.0288 | 0.6905 | 1.6118 | 76.80 | 65.60 | 76.20 |
| **57b** | **5.82** | 4.20 | 15.45 | 19.65 | 1.97351 | 1.88035 | 3.85386 | 0.03196 | 0.0425 | 0.6744 | 1.6375 | 75.36 | 66.72 | 75.53 |
| **58** | **5.91** | 4.38 | 17.70 | 22.08 | 1.9770 | 1.85194 | 3.82894 | 0.03721 | 0.0498 | 0.6570 | 1.6268 | 77.85 | 66.61 | 75.92 |
| **59** | **6.31** | 5.05 | 14.32 | 19.37 | 1.97285 | 1.88715 | 3.86 | 0.03189 | 0.0432 | 0.6675 | 1.6229 | 77.55 | 66.00 | 76.51 |
| **60** | **3.08** | 0.99 | 1.47 | 2.46 | 1.98432 | 1.91718 | 3.9015 | 0.00861 | 0.0057 | 0.7468 | 1.7772 | 74.71 | 66.16 | 71.24 |
| **61** | **3.75** | 1.41 | 2.39 | 3.8 | 1.97684 | 1.92259 | 3.89943 | 0.01037 | 0.0086 | 0.7378 | 1.7242 | 75.12 | 66.62 | 72.18 |
| **62** | **4.14** | 2.75 | 5.98 | 8.73 | 1.97432 | 1.91710 | 3.89142 | 0.01885 | 0.0205 | 0.7174 | 1.7258 | 75.74 | 66.60 | 72.48 |
| **63a** | **5.88** | 4.87 | 6.66 | 11.53 | 1.97342 | 1.89385 | 3.86727 | 0.01824 | 0.0223 | 0.6968 | 1.6381 | 76.65 | 66.18 | 76.10 |
| **63b** | **6.05** | 5.23 | 6.69 | 11.92 | 1.97387 | 1.89020 | 3.86407 | 0.01866 | 0.0232 | 0.6944 | 1.6683 | 76.75 | 66.09 | 74.81 |
| **64a** | **6.20** | 5.96 | 7.82 | 13.78 | 1.97186 | 1.89379 | 3.86565 | 0.02113 | 0.0268 | 0.6862 | 1.6296 | 77.01 | 66.21 | 76.35 |
| **64b** | **6.50** | 5.23 | 8.70 | 13.93 | 1.97447 | 1.89055 | 3.86502 | 0.02142 | 0.0273 | 0.6870 | 1.6569 | 77.00 | 65.64 | 73.41 |
| **64c** | **6.40** | 6.35 | 9.56 | 15.91 | 1.98049 | 1.86417 | 3.84466 | 0.02414 | 0.0319 | 0.6835 | 1.6558 | 77.08 | 66.21 | 74.74 |
| **65** | **4.44** | 2.58 | 2.87 | 5.45 | 1.97477 | 1.88550 | 3.86027 | 0.01917 | 0.0223 | 0.6971 | 1.6756 | 76.62 | 66.13 | 75.09 |
| **66** | **4.03** | 1.71 | 1.98 | 3.69 | 1.97560 | 1.88587 | 3.86147 | 0.01672 | 0.0182 | 0.7032 | 1.6727 | 76.38 | 66.19 | 75.02 |
| **67a** | **6.01** | 5.56 | 10.63 | 16.19 | 1.97160 | 1.89148 | 3.86308 | 0.02491 | 0.0320 | 0.6851 | 1.6302 | 76.98 | 66.30 | 76.36 |
| **67b** | **6.23** | 6.14 | 11.15 | 17.29 | 1.97178 | 1.88775 | 3.85953 | 0.02622 | 0.0343 | 0.6812 | 1.6594 | 77.12 | 66.28 | 75.04 |
| **68a** | **3.71** | 3.84 | 6.00 | 9.84 | 1.97417 | 1.89289 | 3.86706 | 0.02233 | 0.0287 | 0.6901 | 1.6404 | 76.81 | 66.30 | 75.81 |
| **68b** | **4.26** | 5.27 | 8.04 | 13.31 | 1.97325 | 1.88960 | 3.86285 | 0.02682 | 0.0340 | 0.6794 | 1.6620 | 77.17 | 66.16 | 74.77 |
| **69a** | **5.31** | 3.94 | 2.12 | 6.06 | 1.97422 | 1.89684 | 3.87106 | 0.01280 | 0.0142 | 0.7087 | 1.6397 | 76.26 | 66.31 | 76.16 |
| **69b** | **5.30** | 3.99 | 1.58 | 5.57 | 1.97499 | 1.89401 | 3.869 | 0.01250 | 0.0139 | 0.7080 | 1.6694 | 76.30 | 66.13 | 74.97 |
| **70** | **2.03** | – | 1.19 | 1.19 | 1.97979 | 1.91815 | 3.89794 | 0.00782 | 0.0046 | 0.7500 | 1.7085 | 74.51 | 66.44 | 72.88 |
| **71a** | **2.32** | – | 1.26 | 1.26 | 1.97811 | 1.92516 | 3.90327 | 0.00808 | 0.0050 | 0.7495 | 1.7001 | 74.53 | 66.70 | 73.11 |
| **71b** | **2.16** | – | 1.10 | 1.10 | 1.97822 | 1.92528 | 3.9035 | 0.00787 | 0.0047 | 0.7510 | 1.7014 | 74.46 | 66.70 | 73.07 |
| **72a** | **2.61** | – | 1.58 | 1.58 | 1.97786 | 1.92501 | 3.90287 | 0.00821 | 0.0063 | 0.7444 | 1.6956 | 74.93 | 66.69 | 73.26 |
| **72b** | **2.79** | – | 1.89 | 1.89 | 1.97763 | 1.92451 | 3.90214 | 0.00865 | 0.0069 | 0.7422 | 1.6941 | 74.82 | 66.70 | 73.32 |
| **73a** | **2.04** | 0.80 | 2.88 | 3.68 | 1.97589 | 1.92249 | 3.89838 | 0.01157 | 0.0096 | 0.7365 | 1.6815 | 74.95 | 66.91 | 73.70 |
| **73b** | **2.17** | 0.87 | 3.18 | 4.05 | 1.97554 | 1.92150 | 3.89704 | 0.01199 | 0.0103 | 0.7350 | 1.6798 | 75.01 | 66.91 | 73.79 |
| **74** | **2.57** | – | 0.95 | 0.95 | 1.98115 | 1.93141 | 3.91256 | 0.00720 | 0.0040 | 0.7510 | 1.6686 | 74.48 | 65.60 | 71.51 |
| **75** | **2.60** | 0.83 | 3.60 | 4.43 | 1.98127 | 1.93176 | 3.91303 | 0.00672 | 0.0034 | 0.7531 | 1.6699 | 74.40 | 65.68 | 71.38 |
| **76** | **3.22** | – | – | – | 1.97646 | 1.92082 | 3.89728 | 0.00724 | 0.0025 | 0.7620 | 1.6782 | 73.98 | 67.09 | 74.27 |
| **77** | **3.33** | – | – | – | 1.97558 | 1.92109 | 3.89667 | 0.00779 | 0.0031 | 0.7553 | 1.6695 | 74.11 | 67.15 | 74.58 |
| **78** | **2.87** | 0.82 | 1.13 | 1.95 | 1.97573 | 1.92421 | 3.89994 | 0.00819 | 0.0047 | 0.7493 | 1.6890 | 74.58 | 67.07 | 73.38 |
| **79** | **6.56** | 6.50 | 26.44 | 32.94 | 1.97569 | 1.89694 | 3.87263 | 0.04661 | 0.0660 | 0.6476 | 1.7202 | 78.09 | 66.79 | 72.03 |
| **80** | **5.77** | 6.08 | 24.27 | 30.35 | 1.97600 | 1.89834 | 3.87434 | 0.04442 | 0.0617 | 0.6527 | 1.6820 | 77.95 | 65.74 | 73.05 |
| **81a** | **5.25** | 6.55 | 21.39 | 27.94 | 1.97092 | 1.87774 | 3.84866 | 0.04001 | 0.0557 | 0.6588 | 1.5901 | 77.81 | 65.51 | 76.97 |
| **81b** | **5.66** | 7.26 | 23.68 | 30.94 | 1.97066 | 1.87263 | 3.84329 | 0.04348 | 0.0613 | 0.6512 | 1.6178 | 78.05 | 65.42 | 75.79 |
| **82a** | **6.41** | 7.15 | 24.23 | 31.38 | 1.97035 | 1.87534 | 3.84569 | 0.04369 | 0.0621 | 0.6504 | 1.6088 | 78.06 | 65.47 | 76.36 |
| **82b** | **7.01** | 7.98 | 27.21 | 35.19 | 1.96987 | 1.87015 | 3.84002 | 0.04799 | 0.0692 | 0.6415 | 1.6335 | 78.33 | 65.39 | 75.22 |
| **83a** | **9.22** | 9.83 | 36.64 | 46.47 | 1.96507 | 1.87013 | 3.8352 | 0.06097 | 0.0885 | 0.6042 | 1.5343 | 79.62 | 65.58 | – |
| **84a** | **9.20** | 8.86 | 31.55 | 40.41 | 1.96715 | 1.87222 | 3.83937 | 0.05444 | 0.0785 | 0.6189 | 1.5486 | 79.17 | 65.49 | 78.09 |
| **84b** | **10.07** | 9.74 | 35.46 | 45.2 | 1.96651 | 1.86577 | 3.83228 | 0.05970 | 0.0871 | 0.6091 | 1.5747 | 79.45 | 65.42 | 77.01 |
| **85a** | **6.32** | 8.30 | 26.35 | 34.65 | 1.96810 | 1.87702 | 3.84512 | 0.04833 | 0.0682 | 0.6399 | 1.5625 | 78.42 | 65.63 | 77.64 |
| **86a** | **9.61** | 8.11 | 35.32 | 43.43 | 1.96721 | 1.88017 | 3.84738 | 0.05811 | 0.0850 | 0.6224 | 1.4974 | 78.91 | 64.77 | 78.23 |
| **86b** | **9.88** | 8.42 | 37.34 | 45.76 | 1.96689 | 1.87903 | 3.84592 | 0.06025 | 0.0886 | 0.6184 | 1.4965 | 79.03 | 64.71 | 78.63 |
| **87** | **9.02** | 7.80 | 33.88 | 41.68 | 1.96808 | 1.87906 | 3.84714 | 0.05597 | 0.0818 | 0.6264 | 1.5252 | 78.79 | 64.77 | 77.78 |
| **88a** | **4.84** | 4.04 | 8.99 | 13.03 | 1.97529 | 1.91837 | 3.89366 | 0.02817 | 0.0360 | 0.6815 | 1.6792 | 77.07 | 65.64 | 72.01 |
| **88b** | **5.36** | 4.79 | 12.51 | 17.3 | 1.97407 | 1.91367 | 3.88774 | 0.03248 | 0.0430 | 0.6729 | 1.6825 | 77.34 | 65.72 | 72.22 |
| **89** | **6.32** | 5.91 | 17.08 | 22.99 | 1.97621 | 1.90391 | 3.88012 | 0.03592 | 0.0451 | 0.6715 | 1.7477 | 77.36 | 66.20 | 72.41 |
| **90** | **8.13** | 6.32 | 21.87 | 28.19 | 1.97516 | 1.90092 | 3.87608 | 0.04237 | 0.0552 | 0.6558 | 1.6969 | 77.90 | 65.95 | 72.90 |
| **91** | **6.05** | 2.07 | 8.21 | 10.28 | 1.97481 | 1.91600 | 3.89081 | 0.02522 | 0.0293 | 0.6852 | 1.6979 | 76.97 | 66.71 | 73.29 |
| **92** | **8.19** | 5.87 | 28.14 | 34.01 | 1.97020 | 1.90616 | 3.87636 | 0.04782 | 0.0649 | 0.6431 | 1.6581 | 78.25 | 66.46 | 73.49 |
| **93** | **13.36** | 8.80 | 59.71 | 68.51 | 1.96885 | 1.86795 | 3.83680 | 0.08860 | 0.1276 | 0.5696 | 1.5467 | 80.48 | 65.85 | 76.84 |
| **94** | **9.58** | 4.62 | 27.62 | 32.24 | 1.97187 | 1.89937 | 3.87124 | 0.05368 | 0.0731 | 0.6233 | 1.5926 | 78.94 | 66.48 | 75.48 |
| **95** | **12.32** | 9.15 | 33.73 | 42.88 | 1.97561 | 1.84933 | 3.82494 | 0.06929 | 0.0965 | 0.6061 | 1.5913 | 79.38 | 65.89 | 75.39 |
| **96** | **12.07** | 8.11 | 22.78 | 30.89 | 1.97436 | 1.90074 | 3.8751 | 0.05611 | 0.0771 | 0.6325 | 1.6623 | 78.41 | 65.73 | 72.91 |
| **97** | **11.46** | 6.28 | 30.23 | 36.51 | 1.96983 | 1.90035 | 3.87018 | 0.06013 | 0.0848 | 0.6200 | 1.5872 | 78.83 | 66.11 | 75.17 |
| **98** | **11.51** | 8.08 | 32.51 | 40.59 | 1.96636 | 1.89134 | 3.8577 | 0.06696 | 0.0935 | 0.6037 | 1.5758 | 79.42 | 66.14 | 76.14 |
| **99** | **11.83** | 7.97 | 33.06 | 41.03 | 1.96546 | 1.89233 | 3.85779 | 0.06863 | 0.0956 | 0.6006 | 1.5667 | 79.54 | 66.25 | 76.48 |
| **100** | **10.68** | 8.12 | 37.39 | 45.51 | 1.96663 | 1.88600 | 3.85263 | 0.06329 | 0.0892 | 0.6046 | 1.6122 | 79.56 | 66.02 | 75.55 |
| **101** | **12.08** | 8.01 | 33.91 | 41.92 | 1.96579 | 1.89072 | 3.85651 | 0.06780 | 0.0943 | 0.6004 | 1.5886 | 79.60 | 66.12 | 76.03 |
| **102a** | **11.29** | 6.62 | 27.53 | 34.15 | 1.96793 | 1.90512 | 3.87305 | 0.05499 | 0.0732 | 0.6119 | 1.5313 | 79.47 | 66.19 | 77.08 |
| **102b** | **11.76** | 7.16 | 35.17 | 42.33 | 1.96655 | 1.89888 | 3.86543 | 0.06335 | 0.0871 | 0.6013 | 1.5261 | 79.42 | 66.20 | 77.23 |
| **103** | **8.75** | 3.56 | 14.94 | 18.50 | 1.98064 | 1.89544 | 3.87608 | 0.03144 | 0.0427 | 0.6626 | 1.6531 | 77.81 | 65.08 | 73.60 |

^a^The NBO output is truncated.

**Table S10.** The parameters of the linear dependencies D_1_ = A× D_2_ + B of the QTAIM-based descriptors D_1_ on the spectroscopic and structural descriptors D_2_.

| D_1_ | D_2_ | A | B | r |
| --- | --- | --- | --- | --- |
| ρ_BCP_ | ν_O‒H_ | -5·10^‒5^ | 0.203 | 0.958 |
|  | ν_C=O_ | -2·10^‒4^ | 0.308 | 0.493 |
|  | δ_OH_ | 0.003 | 0.009 | 0.900 |
|  | *r*_O∙∙∙H_ | -0.062 | 0.149 | 0.951 |
|  | *r*_O∙∙∙O_ | -1.108 | 0.329 | 0.943 |
|  | *l*_O‒H_ | 1.20 | -1.14 | 0.955 |
|  |  |  |  |  |
| V_BCP_ | ν_O‒H_ | 5·10^‒5^ | -0.22 | 0.961 |
|  | ν_C=O_ | 2·10^‒4^ | -0.349 | 0.512 |
|  | δ_OH_ | -0.003 | -0.005 | 0.891 |
|  | *r*_O∙∙∙H_ | 0.067 | -0.156 | 0.926 |
|  | *r*_O∙∙∙O_ | 0.120 | -0.358 | 0.936 |
|  | *l*_O‒H_ | 1.34 | 1.28 | 0.959 |
|  |  |  |  |  |
| ∇^2^ρ | ν_O‒H_ | -10^‒4^ | 0.525 | 0.903 |
|  | ν_C=O_ | -4·10^‒4^ | 0.777 | 0.461 |
|  | δ_OH_ | 0.008 | 0.053 | 0.897 |
|  | *r*_O∙∙∙H_ | -0.16 | 0.42 | 0.962 |
|  | *r*_O∙∙∙O_ | -0.28 | 0.88 | 0.949 |
|  | *l*_O‒H_ | 2.93 | -2.74 | 0.904 |
|  |  |  |  |  |
| ρ_RCP_ | ν_O‒H_ | 2·10^‒6^ | 0.003 | 0.196 |
|  | ν_C=O_ | -10^‒5^ | 0.032 | 0.145 |
|  | δ_OH_ | -10^‒4^ | 0.013 | 0.121 |
|  | *r*_O∙∙∙H_ | 0.004 | 0.005 | 0.225 |
|  | *r*_O∙∙∙O_ | -4·10^‒4^ | 0.013 | 0.014 |
|  | *l*_O‒H_ | -0.03 | 0.04 | 0.103 |
|  |  |  |  |  |
| ρ_RCP_* | ν_O‒H_ | -10^‒5^ | 0.05 | 0.922 |
|  | ν_C=O_ | -2·10^‒5^ | 0.054 | 0.421 |
|  | δ_OH_ | 5·10^‒4^ | 0.012 | 0.936 |
|  | *r*_O∙∙∙H_ | -0.012 | 0.04 | 0.942 |
|  | *r*_O∙∙∙O_ | -0.022 | 0.075 | 0.962 |
|  | *l*_O‒H_ | 0.23 | -0.20 | 0.914 |

**Table S11.** The parameters of the second order polynomial dependencies D_1_ = A× D_2_^2^ + B× D_2_ + C of the QTAIM-based descriptors D_1_ on the spectroscopic and structural descriptors D_2_.

| D_1_ | D_2_ | A | B | C | r |
| --- | --- | --- | --- | --- | --- |
| ρ_BCP_ | ν_O‒H_ | -3·10^‒8^ | 10^‒4^ | -0.084 | 0.971 |
|  | ν_C=O_ | 10^‒6^ | 0.005 | 4.43 | 0.567 |
|  | δ_OH_ | 2·10^‒4^ | -2·10^‒4^ | 0.018 | 0.922 |
|  | *r*_O∙∙∙H_ | 0.091 | -0.410 | 0.476 | 0.993 |
|  | *r*_O∙∙∙O_ | 0.171 | -1.05 | 1.62 | 0.976 |
|  | *l*_O‒H_ | -7.82 | 16.52 | -8.64 | 0.959 |
|  |  |  |  |  |  |
| V_BCP_ | ν_O‒H_ | 2·10^‒8^ | -9·10^‒5^ | 0.018 | 0.968 |
|  | ν_C=O_ | -2·10^‒6^ | 0.006 | 4.96 | 0.585 |
|  | δ_OH_ | -3·10^‒4^ | 0.001 | -0.016 | 0.924 |
|  | *r*_O∙∙∙H_ | -0.120 | 0.524 | -0.587 | 0.985 |
|  | *r*_O∙∙∙O_ | -0.222 | 1.34 | 2.03 | 0.981 |
|  | *l*_O‒H_ | 4.26 | -9.69 | 5.37 | 0.960 |
|  |  |  |  |  |  |
| ∇^2^ρ | ν_O‒H_ | -10^‒7^ | 7·10^‒4^ | 0.840 | 0.949 |
|  | ν_C=O_ | 3·10^‒6^ | -0.009 | 8.47 | 0.503 |
|  | δ_OH_ | 2·10^‒4^ | 0.005 | 0.06 | 0.899 |
|  | *r*_O∙∙∙H_ | 0.12 | -0.63 | 0.85 | 0.974 |
|  | *r*_O∙∙∙O_ | 0.22 | -1.47 | 2.51 | 0.957 |
|  | *l*_O‒H_ | -56.41 | 113.49 | -56.91 | 0.932 |
|  |  |  |  |  |  |
| ρ_RCP_ | ν_O‒H_ | -3·10^‒9^ | 2·10^‒5^ | 0.031 | 0.210 |
|  | ν_C=O_ | 2·10^‒7^ | -8·10^‒4^ | 0.689 | 0.232 |
|  | δ_OH_ | 10^‒5^ | -3·10^‒4^ | 0.013 | 0.132 |
|  | *r*_O∙∙∙H_ | -0.004 | 0.019 | -0.009 | 0.230 |
|  | *r*_O∙∙∙O_ | 0.012 | 0.066 | -0.078 | 0.073 |
|  | *l*_O‒H_ | 1.15 | -2.29 | 1.15 | 0.144 |
|  |  |  |  |  |  |
| ρ_RCP_* | ν_O‒H_ | -7·10^‒9^ | 4·10^‒5^ | 0.04 | 0.938 |
|  | ν_C=O_ | 3·10^‒7^ | -0.001 | 0.870 | 0.585 |
|  | δ_OH_ | 2·10^‒5^ | 2·10^‒4^ | 0.013 | 0.942 |
|  | *r*_O∙∙∙H_ | 0.012 | -0.058 | 0.083 | 0.959 |
|  | *r*_O∙∙∙O_ | 0.008 | -0.07 | 0.137 | 0.963 |
|  | *l*_O‒H_ | -2.75 | 5.61 | -2.84 | 0.923 |

**Table S12.** The parameters of the linear dependencies D_1_ = A× D_2_ + B of the NBO-based descriptors D_1_ on the spectroscopic, structural and QTAIM-based descriptors D_2_.

| D_1_ | D_2_ | A | B | r |
| --- | --- | --- | --- | --- |
| Σ(σ→σ*) | ρ_BCP_ | 1008.3 | -16.4 | 0,981 |
|  | V_BCP_ | -903.2 | -10.9 | 0,980 |
|  | ∇^2^ρ | 362.2 | -24.68 | 0.944 |
|  | ρ_RCP_ | -1125.7 | 27.25 | 0.270 |
|  | ρ_RCP_* | 3324.1 | 42.06 | 0.829 |
|  | ν_O‒H_ | -0.049 | 188.7 | 0,952 |
|  | ν_C=O_ | -0.158 | 291.6 | 0.478 |
|  | δ_OH_ | 2.90 | -6.93 | 0,887 |
|  | *r*_O∙∙∙H_ | -62.4 | 132.9 | 0,914 |
|  | *r*_O∙∙∙O_ | -106.27 | 307.17 | 0.890 |
|  | *l*_O‒H_ | 1238.1 | -1190.9 | 0,960 |
|  |  |  |  |  |
| n[σ*(O‒H)] | ρ_BCP_ | 1.31 | -0.01 | 0,988 |
|  | V_BCP_ | -1.17 | -0.01 | 0,987 |
|  | ∇^2^ρ | 0.482 | -0.026 | 0.942 |
|  | ρ_RCP_ | -1.42 | 0.04 | 0.260 |
|  | ρ_RCP_* | 4.33 | -0.05 | 0.837 |
|  | ν_O‒H_ | 7·10^‒5^ | 0.26 | 0,979 |
|  | ν_C=O_ | -2·10^‒4^ | 0.40 | 0.507 |
|  | δ_OH_ | 0.004 | -0.002 | 0,900 |
|  | *r*_O∙∙∙H_ | -0.08 | 0.18 | 0,919 |
|  | *r*_O∙∙∙O_ | -0.137 | 0.402 | 0.898 |
|  | *l*_O‒H_ | 1.63 | -1.56 | 0,975 |
|  |  |  |  |  |
| [n(LP_1_)+ n(LP_2_)] | ρ_BCP_ | -1.15 | 3.91 | 0,732 |
|  | V_BCP_ | 0.99 | 3.91 | 0,703 |
|  | ∇^2^ρ | -0.44 | 3.93 | 0.727 |
|  | ρ_RCP_ | 1.61 | 3.86 | 0.251 |
|  | ρ_RCP_* | -4.81 | 3.96 | 0.623 |
|  | ν_O‒H_ | 5·10^‒5^ | 3.68 | 0,693 |
|  | ν_C=O_ | 10^‒5^ | 3.90 | 0.020 |
|  | δ_OH_ | -0.004 | 3.91 | 0,810 |
|  | *r*_O∙∙∙H_ | 0.077 | 3.732 | 0,752 |
|  | *r*_O∙∙∙O_ | 0.12 | 3.55 | 0.665 |
|  | *l*_O‒H_ | -1.41 | 5.26 | 0,715 |
|  |  |  |  |  |
| W(O···H) | ρ_BCP_ | 2.00 | 0.03 | 0,990 |
|  | V_BCP_ | -1.80 | -0.02 | 0,988 |
|  | ∇^2^ρ | 0.74 | -0.048 | 0.946 |
|  | ρ_RCP_ | -2.13 | 0.06 | 0.256 |
|  | ρ_RCP_* | 6.74 | -0.08 | 0.849 |
|  | ν_O‒H_ | 10^‒4^ | 0.36 | 0,976 |
|  | ν_C=O_ | -3·10^‒4^ | 0.60 | 0.493 |
|  | δ_OH_ | 0.006 | -0.011 | 0,910 |
|  | *r*_O∙∙∙H_ | -0.122 | 0.263 | 0,924 |
|  | *r*_O∙∙∙O_ | -0.210 | 0.610 | 0.902 |
|  | *l*_O‒H_ | 142.6 | -62.36 | 0,965 |
|  |  |  |  |  |
| W(O–H) | ρ_BCP_ | -3.32 | 0.80 | 0,977 |
|  | V_BCP_ | 2.96 | 0.78 | 0,969 |
|  | ∇^2^ρ | -1.25 | 0.83 | 0.953 |
|  | ρ_RCP_ | 3.27 | 0.66 | 0.232 |
|  | ρ_RCP_* | -13.15 | 0.92 | 0.868 |
|  | ν_O‒H_ | 2·10^‒4^ | 0.11 | 0,962 |
|  | ν_C=O_ | 5·10^‒4^ | -0.16 | 0.444 |
|  | δ_OH_ | -0.010 | 0.773 | 0,959 |
|  | *r*_O∙∙∙H_ | 0.211 | 0.295 | 0,957 |
|  | *r*_O∙∙∙O_ | 0.356 | -0.285 | 0.910 |
|  | *l*_O‒H_ | -4.19 | 4.78 | 0,972 |
|  |  |  |  |  |
| W(C=O) | ρ_BCP_ | -3.59 | 1.78 | 0,720 |
|  | V_BCP_ | 3.18 | 1.76 | 0,708 |
|  | ∇^2^ρ | -1.37 | 1.82 | 0.710 |
|  | ρ_RCP_ | 1.67 | 1.66 | 0.081 |
|  | ρ_RCP_* | -18.49 | 1.98 | 0.675 |
|  | ν_O‒H_ | 2·10^‒4^ | 1.02 | 0,729 |
|  | ν_C=O_ | 9·10^‒4^ | 0.10 | 0.556 |
|  | δ_OH_ | -0.012 | 1.76 | 0,734 |
|  | *r*_O∙∙∙H_ | 0.22 | 1.26 | 0,664 |
|  | *r*_O∙∙∙O_ | 0.373 | 0.645 | 0.650 |
|  | *l*_O‒H_ | -4.76 | 6.31 | 0,757 |
|  |  |  |  |  |
| P(O–H) | ρ_BCP_ | 112.9 | 73.0 | 0,964 |
|  | V_BCP_ | -100.2 | 73.6 | 0,950 |
|  | ∇^2^ρ | 42.82 | 71.85 | 0.944 |
|  | ρ_RCP_ | -111.2 | 77.6 | 0.231 |
|  | ρ_RCP_* | 529.6 | 67.73 | 0.917 |
|  | ν_O‒H_ | -0.0056 | 96.3 | 0,946 |
|  | ν_C=O_ | -0.0161 | 104.48 | 0.423 |
|  | δ_OH_ | 0.36 | 73.8 | 0,965 |
|  | *r*_O∙∙∙H_ | -7.27 | 90.3 | 0,953 |
|  | *r*_O∙∙∙O_ | -12.58 | 111.08 | 0.907 |
|  | *l*_O‒H_ | 142.6 | -62.4 | 0,965 |
|  |  |  |  |  |
| σ-P(C=O) | ρ_BCP_ | 10.22 | 66.40 | 0,291 |
|  | V_BCP_ | 9.37 | 66.35 | 0,297 |
|  | ∇^2^ρ | -3.88 | 66.51 | 0.285 |
|  | ρ_RCP_ | 4.48 | 66.05 | 0.032 |
|  | ρ_RCP_* | 6.72 | 65.98 | 0.036 |
|  | ν_O‒H_ | 5·10^‒4^ | 64.41 | 0,267 |
|  | ν_C=O_ | 0.0026 | 61.49 | 0.232 |
|  | δ_OH_ | 0.033 | 66.33 | 0,295 |
|  | *r*_O∙∙∙H_ | 0.56 | 65.02 | 0,246 |
|  | *r*_O∙∙∙O_ | 1.16 | 62.89 | 0.287 |
|  | *l*_O‒H_ | 13.52 | 79.95 | 0,305 |
|  |  |  |  |  |
| π-P(C=O) | ρ_BCP_ | 94.49 | 70.95 | 0,635 |
|  | V_BCP_ | 81.71 | 71.54 | 0,609 |
|  | ∇^2^ρ | 36.66 | 69.92 | 0.638 |
|  | ρ_RCP_ | -96.39 | 74.88 | 0.160 |
|  | ρ_RCP_* | 501.0 | 65.51 | 0.646 |
|  | ν_O‒H_ | -0.0046 | 90.26 | 0,620 |
|  | ν_C=O_ | -0.011 | 92.08 | 0.220 |
|  | δ_OH_ | 0.31 | 71.56 | 0,661 |
|  | *r*_O∙∙∙H_ | -6.07 | 85.39 | 0,629 |
|  | *r*_O∙∙∙O_ | -9.68 | 100.5 | 0.567 |
|  | *l*_O‒H_ | 118.2 | -42.2 | 0,639 |
|  |  |  |  |  |

**Table S13.** The parameters of the second order polynomial dependencies D_1_ = A× D_2_^2^ + B× D_2_ + C of the NBO-based descriptors D_1_ on the spectroscopic, structural and QTAIM-based descriptors D_2_.

| D_1_ | D_2_ | A | B | C | r |
| --- | --- | --- | --- | --- | --- |
| Σ(σ→σ*) | ρ_BCP_ | 7595.8 | 452.3 | -8.0 | 0,987 |
|  | V_BCP_ | 3275.5 | -667.9 | -7.7 | 0,982 |
|  | ∇^2^ρ | 2851 | -286.0 | 8.50 | 0.975 |
|  | ρ_RCP_ | 395616 | 10864 | 82.6 | 0.499 |
|  | ρ_RCP_* | 506086 | -12317 | 76.0 | 0.973 |
|  | ν_O‒H_ | 2·10^‒5^ | 0.072 | 11.59 | 0,958 |
|  | ν_C=O_ | 0.002 | -5.33 | 4801.5 | 0.563 |
|  | δ_OH_ | 0.34 | -2.46 | 7.47 | 0,947 |
|  | *r*_O∙∙∙H_ | 128.0 | -548.4 | 588.8 | 0,987 |
|  | *r*_O∙∙∙O_ | 240.38 | -1428.3 | 2126.6 | 0.955 |
|  | *l*_O‒H_ | 911.0 | -547.5 | -316.1 | 0,960 |
|  |  |  |  |  |  |
| n[σ*(O‒H)] | ρ_BCP_ | 8.61 | 0.68 | 0.01 | 0,993 |
|  | V_BCP_ | 2.83 | -0.97 | -0.01 | 0,988 |
|  | ∇^2^ρ | 3.75 | -0.367 | 0.167 | 0.973 |
|  | ρ_RCP_ | 539.4 | -14.7 | 0.117 | 0.512 |
|  | ρ_RCP_* | 631.4 | -15.8 | 0.10 | 0.971 |
|  | ν_O‒H_ | -2·10^‒8^ | 7·10^‒5^ | 0.04 | 0,983 |
|  | ν_C=O_ | 10^‒6^ | -0.0069 | 6.27 | 0.589 |
|  | δ_OH_ | 0.0004 | -0.0024 | 0.014 | 0,952 |
|  | *r*_O∙∙∙H_ | 0.16 | -0.67 | 0.74 | 0,992 |
|  | *r*_O∙∙∙O_ | 0.304 | -1.81 | 2.70 | 0.960 |
|  | *l*_O‒H_ | -1.50 | 4.57 | -3.00 | 0,975 |
|  |  |  |  |  |  |
| [n(LP_1_)+ n(LP_2_)] | ρ_BCP_ | 17.41 | -2.41 | 3.93 | 0,749 |
|  | V_BCP_ | 12.59 | 1.89 | 3.92 | 0,720 |
|  | ∇^2^ρ | 0.029 | -0.448 | 3.93 | 0.727 |
|  | ρ_RCP_ | -529.6 | 14.66 | 3.79 | 0.442 |
|  | ρ_RCP_* | -337.2 | 5.44 | 3.88 | 0.650 |
|  | ν_O‒H_ | 8·10^‒8^ | 0.001 | 4.615 | 0,768 |
|  | ν_C=O_ | -2·10^‒6^ | 0.0075 | -2.69 | 0.285 |
|  | δ_OH_ | 6·10^‒5^ | 0.005 | 3.911 | 0,810 |
|  | *r*_O∙∙∙H_ | -0.040 | 0.229 | 3.589 | 0,756 |
|  | *r*_O∙∙∙O_ | -0.34 | 0.860 | 2.53 | 0.677 |
|  | *l*_O‒H_ | 39.72 | -79.25 | 43.39 | 0,762 |
|  |  |  |  |  |  |
| W(O···H) | ρ_BCP_ | 11.04 | 1.20 | -0.02 | 0,993 |
|  | V_BCP_ | 2.62 | -1.61 | -0.02 | 0,988 |
|  | ∇^2^ρ | 5.52 | -0.510 | 0.016 | 0.975 |
|  | ρ_RCP_ | 816.0 | -22.22 | 0.17 | 0.505 |
|  | ρ_RCP_* | 909.6 | -21.36 | 0.13 | 0.967 |
|  | ν_O‒H_ | -4·10^‒8^ | -2·10^‒4^ | 0.04 | 0,983 |
|  | ν_C=O_ | 3·10^‒6^ | -0.40 | 9.42 | 0.575 |
|  | δ_OH_ | 6·10^‒4^ | 0.003 | 0.013 | 0,957 |
|  | *r*_O∙∙∙H_ | 0.232 | 1.010 | 1.100 | 0,993 |
|  | *r*_O∙∙∙O_ | 0.451 | -2.693 | 4.02 | 0.960 |
|  | *l*_O‒H_ | -6.05 | 14.34 | 8.20 | 0,977 |
|  |  |  |  |  |  |
| W(O–H) | ρ_BCP_ | 13.70 | -4.33 | 0.81 | 0,979 |
|  | V_BCP_ | 18.12 | 4.31 | 0.80 | 0,975 |
|  | ∇^2^ρ | -4.25 | -0.290 | 0.78 | 0.960 |
|  | ρ_RCP_ | -1470 | 39.5 | 0.45 | 0.520 |
|  | ρ_RCP_* | -1420 | 30.7 | 0.59 | 0.947 |
|  | ν_O‒H_ | 10^‒7^ | -7·10^‒4^ | 1.60 | 0,993 |
|  | ν_C=O_ | 5·10^‒6^ | 0.019 | -15.92 | 0.542 |
|  | δ_OH_ | -5·10^‒4^ | -3·10^‒4^ | 0.75 | 0,969 |
|  | *r*_O∙∙∙H_ | -0.25 | 1.17 | -0.60 | 0,985 |
|  | *r*_O∙∙∙O_ | -0.578 | 3.54 | -4.66 | 0.944 |
|  | *l*_O‒H_ | 55.24 | -112.46 | 57.81 | 0,987 |
|  |  |  |  |  |  |
| W(C=O) | ρ_BCP_ | -19.94 | -2.14 | 1.76 | 0,722 |
|  | V_BCP_ | -9.75 | 2.48 | 1.75 | 0,709 |
|  | ∇^2^ρ | -8.53 | 0.56 | 1.72 | 0.726 |
|  | ρ_RCP_ | -1432 | 36.9 | 1.46 | 0.320 |
|  | ρ_RCP_* | -821.9 | 6.48 | 1.79 | 0.687 |
|  | ν_O‒H_ | 10^‒7^ | 7·10^‒4^ | 2.48 | 0,747 |
|  | ν_C=O_ | -5·10^‒6^ | 0.018 | -15.08 | 0.593 |
|  | δ_OH_ | -8·10^‒4^ | -7·10^‒5^ | 1.73 | 0,750 |
|  | *r*_O∙∙∙H_ | -0.39 | 1.72 | -0.15 | 0,710 |
|  | *r*_O∙∙∙O_ | -0.83 | 4.94 | -5.64 | 0.695 |
|  | *l*_O‒H_ | 21.88 | -47.64 | 27.32 | 0,759 |
|  |  |  |  |  |  |
| P(O–H) | ρ_BCP_ | -903.3 | 178.7 | 72.0 | 0,971 |
|  | V_BCP_ | -877.1 | -163.0 | 72.8 | 0,961 |
|  | ∇^2^ρ | 89.53 | 22.53 | 72.87 | 0.946 |
|  | ρ_RCP_ | 52.53 | -1405 | 85.0 | 0.536 |
|  | ρ_RCP_* | 32224 | -494 | 75.7 | 0.937 |
|  | ν_O‒H_ | -6·10^‒6^ | 0.032 | 34.30 | 0,991 |
|  | ν_C=O_ | 2·10^‒4^ | -0.654 | 661.2 | 0.532 |
|  | δ_OH_ | 0.009 | 0.22 | 74.2 | 0,968 |
|  | *r*_O∙∙∙H_ | 6.42 | -31.8 | 113.4 | 0,969 |
|  | *r*_O∙∙∙O_ | 16.94 | -105.48 | 238.18 | 0.925 |
|  | *l*_O‒H_ | -2343.1 | 4734.9 | 2312.1 | 0,987 |
|  |  |  |  |  |  |
| σ-P(C=O) | ρ_BCP_ | -125.98 | -1.07 | 66.27 | 0,296 |
|  | V_BCP_ | -19.25 | 7.99 | 66.34 | 0,297 |
|  | ∇^2^ρ | -54.47 | 8.47 | 65.88 | 0.316 |
|  | ρ_RCP_ | -144.4 | 8.04 | 66.03 | 0.032 |
|  | ρ_RCP_* | 274.6 | -1.62 | 66.04 | 0.036 |
|  | ν_O‒H_ | 9·10^‒7^ | 0.0055 | 74.24 | 0,309 |
|  | ν_C=O_ | -3·10^‒5^ | 0.099 | -22.44 | 0.282 |
|  | δ_OH_ | -0.0021 | -7·10^‒4^ | 66.25 | 0,301 |
|  | *r*_O∙∙∙H_ | 1.91 | 7.87 | 58.13 | 0,301 |
|  | *r*_O∙∙∙O_ | 0.66 | 4.79 | 57.90 | 0.289 |
|  | *l*_O‒H_ | 177.5 | -361.5 | 249.7 | 0,310 |
|  |  |  |  |  |  |
| π-P(C=O) | ρ_BCP_ | -704.27 | 145.49 | 70.19 | 0,638 |
|  | V_BCP_ | -469.58 | -115.19 | 71.09 | 0,612 |
|  | ∇^2^ρ | 34.43 | 28.89 | 70.31 | 0.638 |
|  | ρ_RCP_ | 41988 | -1130 | 80.77 | 0.348 |
|  | ρ_RCP_* | 21648 | -156.8 | 70.4 | 0.657 |
|  | ν_O‒H_ | 6·10^‒6^ | 0.034 | 26.38 | 0,665 |
|  | ν_C=O_ | 10^‒4^ | -0.53 | 544.9 | 0.303 |
|  | δ_OH_ | 0.010 | 0.15 | 71.97 | 0,665 |
|  | *r*_O∙∙∙H_ | 4.65 | 23.85 | 102.17 | 0,637 |
|  | *r*_O∙∙∙O_ | 15.40 | -94.50 | 217.1 | 0.587 |
|  | *l*_O‒H_ | -1917 | 3878.3 | -1884.8 | 0,662 |
|  |  |  |  |  |  |

**Table S14.** The parameters of the linear dependencies D = A× [‒*E*_HB_(MTA)] + B and the second order polynomial dependencies D = A× [‒*E*_HB_(MTA)]^2^ + B× [‒*E*_HB_(MTA)] + C of the spectroscopic, structural, QTAIM-based and NBO-based descriptors D on the ‒*E*_HB_(MTA) hydrogen bond energy estimated via molecular tailoring approach.

| type of  descriptor | descriptor | linear | | | second-order polynomial | | | | Δr |
| --- | --- | --- | --- | --- | --- | --- | --- | --- | --- |
|  |  | A | B | r | A | B | C | r |  |
| spectroscopic | ν_O‒H_ | -90.33 | 4009.1 | 0,960 | -2.14 | -61.22 | 3935.3 | 0,962 | 0,002 |
|  | ν_C=O_ | -8.69 | 1797.4 | 0,587 | -1.26 | 8.41 | 1754.1 | 0,644 | 0,057 |
|  | δ_OH_ | 1.37 | 0.25 | 0,892 | -0.12 | 2.97 | -3.82 | 0,926 | 0,034 |
| structural | *r*_O∙∙∙H_ | -0.062 | 2.23 | 0,854 | 0.005 | -0.138 | 2.42 | 0,888 | 0,034 |
|  | *l*_O‒H_ | 0.004 | 0.955 | 0,964 | 2·10^‒5^ | 0.003 | 0.955 | 0,964 | 0 |
|  | *r*_O∙∙∙O_ | -0.034 | 2.93 | 0,819 | 7·10^‒4^ | -0.043 | 2.96 | 0,821 | 0,002 |
| QTAIM-based | ρ_BCP_ | 0.004 | 0.008 | 0,916 | 6·10^‒5^ | 0.005 | 0.006 | 0,917 | 0,001 |
|  | V_BCP_ | -0.005 | -0.003 | 0,917 | -10^‒5^ | -0.005 | -0.003 | 0,917 | 0 |
|  | ∇^2^ρ | 0.011 | 0.051 | 0,869 | -6·10^‒4^ | 0.019 | 0.030 | 0,883 | 0,014 |
|  | ρ_RCP_ | -2·10^‒4^ | 0.013 | 0,157 | 6·10^‒5^ | -0.001 | 0.015 | 0,230 | 0,073 |
|  | ρ_RCP_* | 8·10^‒4^ | 0.012 | 0,864 | -3·10^‒5^ | 0.001 | 0.04 | 0,873 | 0,009 |
| NBO-based | Σ(σ→σ*) | 4.47 | -8.65 | 0,917 | 0.002 | 4.45 | -8.59 | 0,917 | 0 |
|  | n[σ*(O‒H)] | 0.006 | -0.006 | 0,939 | 2·10^‒5^ | 0.006 | -0.004 | 0,940 | 0,001 |
|  | [n(LP_1_)+n(LP_2_)] | -0.005 | 3.90 | 0,652 | 0.0007 | -0.014 | 3.93 | 0,714 | 0,062 |
|  | W(O···H) | 0.009 | -0.015 | 0,938 | -4·10^‒5^ | 0.010 | -0.016 | 0,938 | 0 |
|  | W(O–H) | -0.015 | 0.78 | 0,938 | 0.0007 | -0.025 | 0.80 | 0,948 | 0,010 |
|  | W(C=O) | -0.018 | 1.77 | 0,757 | 8·10^‒4^ | -0.029 | 1.79 | 0,765 | 0,008 |
|  | P(O–H) | 0.52 | 73.73 | 0,928 | -0.030 | 0.93 | 72.70 | 0,944 | 0,016 |
|  | σ-P(C=O) | -0.055 | 66.38 | 0,328 | 0.007 | -0.143 | 66.60 | 0,350 | 0,022 |
|  | π-P(C=O) | 0.44 | 71.55 | 0,622 | -0.041 | 0.992 | 70.16 | 0,648 | 0,026 |
